# Genetic risk scores for major psychiatric disorders and the risk of postpartum psychiatric disorders

**DOI:** 10.1038/s41398-019-0629-9

**Published:** 2019-11-11

**Authors:** Anna E. Bauer, Xiaoqin Liu, Enda M. Byrne, Patrick F. Sullivan, Naomi R. Wray, Esben Agerbo, Mette Nyegaard, Jakob Grove, Katherine L. Musliner, Katja G. Ingstrup, Benedicte M. W. Johannsen, Merete L. Mægbæk, Yunpeng Wang, Merete Nordentoft, Ole Mors, Anders D. Børglum, Thomas Werge, David M. Hougaard, Preben Bo Mortensen, Trine Munk-Olsen, Samantha Meltzer-Brody

**Affiliations:** 1 Department of Psychiatry, University of North Carolina School of Medicine, Chapel Hill, NC USA; 2 NCRR - National Centre for Register-Based Research, Aarhus University, Aarhus, Denmark; 3 iPSYCH, Lundbeck Foundation Initiative for Integrative Psychiatric Research, Aarhus, Denmark; 4 Institute for Molecular Bioscience, University of Queensland, Brisbane, QLD Australia; 5 Department of Genetics, University of North Carolina School of Medicine, Chapel Hill, NC USA; 6 Department of Medical Epidemiology and Biostatistics, Karolinska Institutet, Stockholm, Sweden; 7 Queensland Brain Institute, University of Queensland, Brisbane, QLD Australia; 8 CIRRAU - Centre for Integrated Register-Based Research, Aarhus University, Aarhus, Denmark; 9 Department of Biomedicine, Aarhus University, Aarhus, Denmark; 10 iSEQ, Center for Integrative Sequencing and, Center for Genomics and Personalized Medicine, Aarhus, Denmark; 11 Bioinformatics Research Centre, Aarhus University, Aarhus, Denmark; 12 Institute of Biological Psychiatry, Mental Health Center Sct. Hans, Mental Health Services Capital Region of Denmark, Copenhagen, Denmark; 13 Lifespan Changes in Brain and Cognition (LCBC), Department of Psychology, University of Oslo, Oslo, Norway; 14 Mental Health Centre Copenhagen, Copenhagen, Denmark; 15 Institute of Clinical Medicine, University of Copenhagen, Copenhagen, Denmark; 16 Psychosis Research Unit, Aarhus University Hospital, Risskov, Aarhus, Denmark; 17 Danish Center for Neonatal Screening, Department for Congenital Disorders, Statens Serum Institut, Copenhagen, Denmark

**Keywords:** Depression, Bipolar disorder, Predictive markers, Genomics

## Abstract

Postpartum psychiatric disorders are heritable, but how genetic liability varies by other significant risk factors is unknown. We aimed to (1) estimate associations of genetic risk scores (GRS) for major depression (MD), bipolar disorder (BD), and schizophrenia (SCZ) with postpartum psychiatric disorders, (2) examine differences by prior psychiatric history, and (3) compare genetic and familial risk of postpartum psychiatric disorders. We conducted a nested case-control study based on Danish population-based registers of all women in the iPSYCH2012 cohort who had given birth before December 31, 2015 (*n* = 8850). Cases were women with a diagnosed psychiatric disorder or a filled psychotropic prescription within one year after delivery (*n* = 5829 cases, 3021 controls). Association analyses were conducted between GRS calculated from Psychiatric Genomics Consortium discovery meta-analyses for MD, BD, and SCZ and case-control status of a postpartum psychiatric disorder. Parental psychiatric history was associated with postpartum psychiatric disorders among women with previous psychiatric history (OR, 1.14; 95% CI 1.02–1.28) but not without psychiatric history (OR, 1.08; 95% CI: 0.81–1.43). GRS for MD was associated with an increased risk of postpartum psychiatric disorders in both women with (OR, 1.44; 95% CI: 1.19–1.74) and without (OR, 1.88; 95% CI: 1.26–2.81) personal psychiatric history. SCZ GRS was only minimally associated with postpartum disorders and BD GRS was not. Results suggest GRS of lifetime psychiatric illness can be applied to the postpartum period, which may provide clues about distinct environmental or genetic elements of postpartum psychiatric disorders and ultimately help identify vulnerable groups.

## Introduction

The postpartum period is one of the most vulnerable times for psychiatric illness in a woman’s life^1–3^ and is associated with a highly increased risk of depression and other disorders^3–8^. Postpartum psychiatric disorders vary in prevalence, severity, and timing of onset, and have a wide spectrum of clinical symptoms^9–13^. They include unipolar depression, which is common and occurs in ~10–15% of postpartum women^14^ and more rare and severe disorders like bipolar disorder occurring in 3% of postpartum women^15^, and postpartum psychosis that occurs in 1 to 2 per 1000 in the first few weeks after delivery^3,16,17^. Personal^2,4–6,18,19^ and family history^20–26^ of psychiatric illness are two of the strongest risk factors for postpartum psychiatric disorders. A strong association between family history and postpartum psychiatric disorders suggests genetic vulnerability for these disorders, which has been confirmed in a recent heritability study of perinatal depression^27^. Moreover, heritability of postpartum depression may be greater than that of major depression outside of the perinatal period (44% versus 32%)^27^.

Genetic liability can be estimated as genetic risk scores (GRS), a score produced for each individual by weighting the effect size of multiple risk alleles obtained from genetic association studies^28^. Increasingly robust findings from genetic association studies have been used to construct GRS for major depression (MD)^29^, bipolar disorder (BD)^30^, and schizophrenia (SCZ)^31^ and evaluate shared genetic risk between psychiatric traits^32–34^. Thus far, a single study has considered GRS and risk of postpartum depression and found that BD GRS was more strongly associated with postpartum depression than major depression outside the postpartum period (*R*^*2*^ *=* 1.64%, *p* = 3.04 × 10^−5^ versus *R*^*2*^ *=* 0.4%, *p* = 0.02)^35^. While genetic risk scores have been applied to postpartum psychiatric disorders to evaluate genetic overlap with other psychiatric disorders^35^, they have not been used to explore how these correlations differ by other risk factors. Assessing how genetic liability varies by specific and significant risk factors may help identify subgroups with shared etiology.

We aimed to determine whether genetic risk scores for MD, BD, and SCZ predict psychiatric disorders occurring specifically in the postpartum period. Building on prior work, we also sought to examine how risk score predictions differ in women with a prior psychiatric history or without, and how genetic risks compare to risks associated with family history of psychiatric disorders.

## Materials and methods

### Study design

**Fig. 1:**
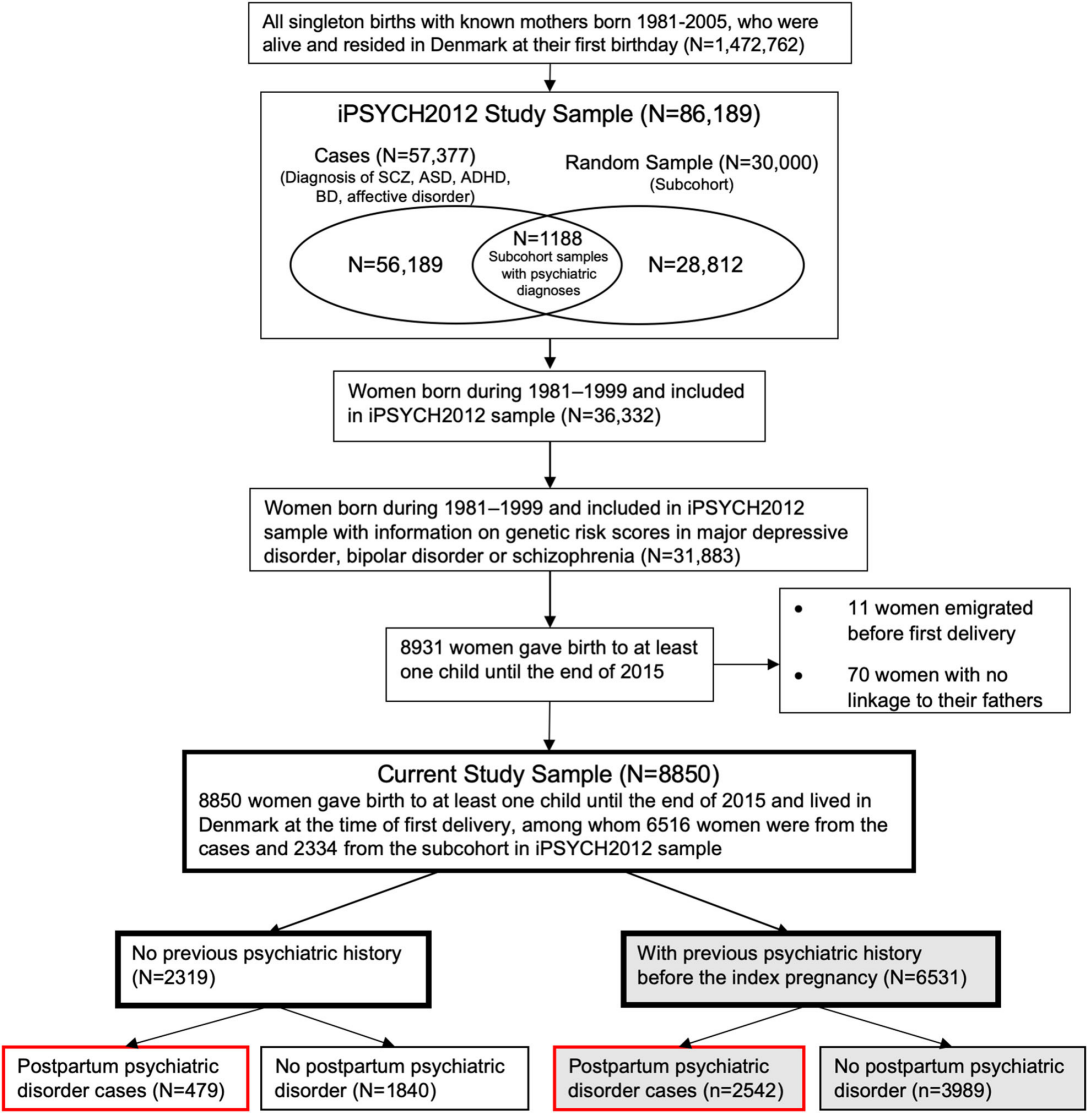
Flowchart illustrating the identification of the study population.

Data from Danish population-based registers were linked by unique personal identification numbers, which are assigned to all live-born children and new residents in Denmark and enable linkage across all national registers. We conducted a case-control study selected from the larger iPSYCH2012 study sample, for which sample selection^36^ and genetic analytic methods^34^ have been described elsewhere (Supplementary Methods). Briefly, the iPSYCH2012 sample was selected from the Danish Civil Registration System^36^ of all singleton births born between May 1st, 1981 and December 31st, 2005 who were alive and resided in Denmark at one year of age and whose mother was known. The full cohort was then linked to the Danish Psychiatric Central Research Register^37^. All subjects within the full cohort who had a diagnosis of SCZ, autism spectrum disorder, attention-deficit/hyperactivity disorder, BD, and affective disorder were identified as cases in the iPSYCH2012 sample (*N* = 57,377). A random sample of 30,000 subjects (i.e., the subcohort), was selected from the full cohort from which the cases were drawn (Fig. 1)^36^. This random sample represents a population sample to be used as controls in the genetic analysis. However, since these 30,000 subjects were chosen randomly from the full cohort, some also have psychiatric disorders.

### Study population

For the current study, eligible study subjects were all women born during 1981–1999 and included in the iPSYCH2012 study sample who gave birth to at least one child before December 31st, 2015 and passed genetic quality control (*N* = 8931) (Fig. 1). We excluded 11 women who emigrated before their first delivery and 70 women that could not be linked to their fathers in the register. Altogether 8850 women were included in our final analysis.

### Postpartum psychiatric disorders

We defined a postpartum psychiatric disorder either as (1) a treated psychiatric episode recorded in the Danish Psychiatric Central Research Register or (2) at least one redeemed prescription of psychotropic drugs recorded in the Danish National Prescription Registry within 12 months after delivery. Consequently, our case group consisted of moderate (prescription defined) and severe (treated in secondary health care and diagnosed by a psychiatrist) cases (Supplementary Table S1). We selected the time period of interest as one year after childbirth to account for a potential delay in seeking psychiatric care and to apply a widely used clinical definition^38^. Prior work in these data showed similar familial risk of postpartum psychiatric disorders at 3, 6, and 12 months postpartum^39^. A postpartum psychiatric episode may represent a new-onset disorder, a continuation or recurrence of a psychiatric disorder prior to childbirth, or a different psychiatric disorder after childbirth in a woman who had a prior psychiatric disorder.

The Danish Psychiatric Central Research Register contains information on inpatient contacts at psychiatric hospitals and psychiatric wards from 1969 onwards. Psychiatric outpatient treatment and emergency room contacts have been included since 1995^37^. Treated psychiatric episodes included any psychiatric diagnosis (Primary diagnosis, ICD-10 codes F00–F99), excluding mental retardation and substance abuse (ICD-10 codes F10–F19 and F70–F79). The Danish National Prescription Registry was established in 1995, and contains information on all prescriptions dispensed at community pharmacies in Denmark^40^. We included psychotropic drugs of Anatomical Therapeutic Chemical (ATC) classification codes N05 (psycholeptics) and N06 (psychoanaleptics). Detailed diagnostic codes and categorization are described in the Supplementary Methods.

### Personal and parental psychiatric history

We defined previous personal psychiatric history as one in-patient or out-patient treatment for the psychiatric disorders described above or one redeemed prescription for psychotropic medications before the date of delivery. We similarly defined parental psychiatric history as one in-patient or out-patient treatment for psychiatric disorders in a mother or father before the date of delivery. Note, as information on prescriptions only dates back to 1995, prescription data were not used to define parental psychiatric history.

### Genotyping, quality control, and imputation

The Danish Newborn Screening Biobank stored dried blood spots taken at birth from nearly all infants born in Denmark since May 1st 1981^41^. Genetic data were extracted from the dried blood spot samples stored in this biobank, whole-genome amplified (in triplicate using the Qiagen REPLI-g mini kit and the three separate reactions were pooled), and genotyped with Illumina Infinium HD Human610-Quad BeadChip^42^. Information about non-genotyped markers was obtained by imputation using the 1000 genomes phase 3 as reference panel. Quality control and imputation were conducted using the Ricopili pipeline^43^ (Supplementary Methods).

### Genetic risk scores

We calculated genetic risk scores for MD^29^, BD^30^, and SCZ^44^ based on genome-wide data from Psychiatric Genomics Consortium (PGC) discovery sample excluding individuals in the iPSYCH2012 sample. We selected single nucleotide polymorphisms (SNPs) associated with MD, BP, and SCZ at a *P*-value threshold of 0.05 or lower, which were reported as maximising out-of-sample prediction into multiple cohorts of the study specific disorders (Supplementary Table S2)^44–46^ and generated the polygenic risk score for each individual based on this threshold (See Grove et al.^34^ and Supplementary Methods for details).

### Statistical analysis

Chi-squared tests were used to evaluate differences in characteristics between cases and controls in the overall cohort and among women with or without prior psychiatric history, except in the case of maternal age at index delivery, in which age distributions were compared with *t*-tests. Binary logistic regression models were used to estimate the odds ratios (ORs) of postpartum psychiatric disorders with 95% confidence intervals (CIs) using Stata 13.1. We converted genetic risk scores into z-scores according to the means and standard deviations from the distributions in women born during 1981–1999 from the subcohort based on the following formula: (observed value − mean)/standard deviation. We analyzed standardized genetic risk scores as continuous variables, reported as a unit change for one standard deviation or 10 deciles, and categorically by decile with the lowest decile as the reference group. To account for ancestry differences, we adjusted for the first four principal components estimated from genome-wide SNP genotypes in all models^47^. To address the possible secular trends in diagnostic practices and to account for the fact that fewer blood spots were retrievable among women born in the earlier years, we also included woman's calendar year of birth as a covariate in all models. In the adjusted models, we included parental psychiatric history before the index delivery, parental country of origin, primiparity, age and age-squared at the index delivery as additional covariates. Because approximately 9% of the sample had a parent born outside of Denmark, and thus, may be of mixed ancestry, we conducted a sensitivity analysis restricting to women only with parents of Danish origin. We present results in the overall cohort but also stratified by women with or without psychiatric history prior to childbirth, as first onset and recurrent postpartum psychiatric disorders may reflect different phenotypes. We tested for statistical interaction between GRS and psychiatric history to assess whether the associations between GRS and postpartum psychiatric disorders were modified by previous psychiatric history. To account for multiple corrections, we applied a Bonferroni corrected *P*-value of 0.017 for α = 0.05 and three independent tests (MD, BD, and SCZ GRS). We computed area under the covariate-adjusted receiver-operator characteristic (ROC) curve to evaluate the ability of the GRS for MD, BD, and SCZ to predict postpartum psychiatric disorder case status.

Our primary outcome of interest was a psychiatric disorder in the postpartum period, but to test to which extent genetic risk scores in general predict psychiatric disorders in our sample, we also conducted an analysis in which cases consisted of psychiatric disorders at any time point and controls were individuals free of psychiatric disorders at any time.To determine whether results were influenced by the *P*-value threshold used to select SNPs from the discovery sample for inclusion in the risk score, we repeated our analysis using all SNPs (*P*_T_ ≤ 1). Because our broad case definition might also influence results, we conducted a sensitivity analysis in which we categorized postpartum psychiatric disorders into two mutually exclusive subsets defined by severity of the postpartum disorder: (1) hospital contact regardless of psychotropic treatment and (2) only psychotropic medication use.

### Ethics

The study was approved by the Danish Scientific Ethical Committee System, the Danish Data Protection Agency, and the Danish Neonatal Screening Biobank Steering Committee. By Danish law, no informed consent is required for a register-based study on the basis of anonymized data. The study was also approved by the Institutional Review Board at the University of North Carolina at Chapel Hill.

## Results

The final dataset consisted of 8850 women who had at least one recorded childbirth. Median age was 25.0 (interquartile range: 23.0–28.8) years and the majority were primiparous (64.4%). The sample included 479 postpartum psychiatric disorder cases and 1840 non-cases among women with no previous personal psychiatric history, and 2542 postpartum psychiatric disorder cases and 3989 non-cases among women with previous personal psychiatric history (Table 1). Cases in the sample consisted predominantly of those defined by unipolar depression diagnosis or redeemed prescription for antidepressants; 343 of 479 cases (71.6%) among women with no previous personal psychiatric history, and 1540 of 2542 cases (60.6%) among women with previous personal psychiatric history had either in- or outpatient treatment for depression or redeemed prescription for antidepressants. Compared to non-cases, cases of postpartum psychiatric disorders were more likely to have parental psychiatric history (*P* < 0.001) and to be younger (*P* < 0.001) and primiparous (*P* < 0.001).

**Table 1:** Characteristics of the study population

	Full cohort (*N* = 8850)			Women with no previous psychiatric history (*N* = 2319)			Women with previous psychiatric history (*N* = 6531)			
Characteristics	Cases (*N* = 3021)	Non-cases (*N* = 5829)	*P*-value	Cases of postpartum psychiatric disorders (*N* = 479)	Non-cases (*N* = 1840)	*P*-value	Cases of postpartum psychiatric disorders (*N* = 2542)	Non-cases (*N* = 3989)	*P*-value	*P*-value for cases between strata
Subtypes of postpartum psychiatric disorders										
Schizophrenia diagnosis	78 (2.6)	–		10 (2.1)	–		68 (2.7)	–		
Bipolar disorder diagnosis	41 (1.4)	–		4 (0.8)	–		37 (1.5)	–		
Unipolar depression diagnosis	511 (16.9)	–		186 (38.8)	–		325 (12.8)	–		
Neurotic, stress-related, and somatoform disorder	288 (9.5)	–		57 (11.9)	–		231 (9.1)	–		
Other psychiatric diagnosis	370 (12.2)	–		36 (7.5)	–		334 (13.1)	–		
Antidepressant treatment	1 372 (45.4)	–		157 (32.8)	–		1 215 (47.8)	–		
Other psychotropic medication treatment	361 (11.9)	–		29 (6.1)	–		332 (13.1)	–		
Parental psychiatric history before delivery	881 (29.2)	1 429 (24.5)	<0.001	101 (21.1)	292 (15.9)	0.007	780 (30.7)	1 137 (28.5)	0.059	<0.001
Parental country of origin										
Denmark	2 756 (91.2)	5 270 (90.4)	0.209	432 (90.2)	1 676 (91.1)	0.542	2 324 (91.4)	3 594 (90.1)	0.073	0.380
At least one parent outside Denmark	265 (8.8)	559 (9.6)		47 (9.8)	164 (8.9)		218 (8.6)	395 (9.9)		
Age at the index delivery (years), mean ± SD	24.8 ± 3.6	26.4 ± 3.8	<0.001	23.1 ± 3.2	26.4 ± 4.0	<0.001	25.2 ± 3.5	26.4 ± 3.7	<0.001	<0.001
Primiparous										
Yes	2 342 (77.5)	3 354 (57.5)	<0.001	344 (71.8)	962 (52.3)	<0.001	1 998 (78.6)	2 392 (60.0)	<0.001	0.001
No	679 (22.5)	2 475 (42.5)		135 (28.2)	878 (47.7)		544 (21.4)	1 597 (40.0)		
Calendar birth year of the woman										
1981–1985	1 699 (56.2)	2 933 (50.3)	<0.001	298 (62.2)	1 076 (58.5)	0.199	1 401 (55.1)	1 857 (46.5)	<0.001	<0.001
1986–1990	1 069 (35.4)	2 184 (37.5)		156 (32.6)	634 (34.5)		913 (35.9)	1 550 (38.9)		
1991–1999	253 (8.4)	712 (12.2)		25 (5.2)	130 (7.1)		228 (9.0)	582 (14.6)		

After controlling for potential confounders, parental psychiatric history was associated with a higher risk of postpartum psychiatric disorders among women with previous personal psychiatric history (OR: 1.14, 95% CI: 1.02–1.28) but not among women without personal psychiatric history (OR: 1.08, 95% CI: 0.81–1.43). An increased risk was observed in primiparous women, OR: 2.42 (95% CI: 2.14–2.74) and 1.63 (95% CI: 1.27–2.11) for women with and without prior personal psychiatric history, respectively (Table 2) (Full model, Supplementary Table S3).

**Table 2:** Odds ratio for postpartum psychiatric disorders during the follow-up period according to baseline characteristics

Characteristics	Full cohort		Women with no previous psychiatric history		Women with previous psychiatric history	
	Crude OR^a^ (95% CI)	Adjusted OR^a,b^ (95% CI)	Crude OR^a^ (95% CI)	Adjusted OR^a,b^ (95% CI)	Crude OR^a^ (95% CI)	Adjusted OR^a,b^ (95% CI)
Parental psychiatric history						
No	1 (ref)	1 (ref)	1 (ref)	1 (ref)	1 (ref)	1 (ref)
Yes	1.31 (1.19–1.45)	1.27 (1.14–1.41)	1.43 (1.11–1.84)	1.08 (0.81–1.43)	1.16 (1.04–1.30)	1.14 (1.02–1.28)
Parental country of origin						
Denmark	1 (ref)	1 (ref)	1 (ref)	1 (ref)	1 (ref)	1 (ref)
At least one parent outside Denmark	1.05 (0.86–1.27)	1.05 (0.85–1.29)	1.48 (0.94–2.31)	1.43 (0.86–2.36)	0.92 (0.74–1.15)	0.95 (0.76–1.20)
Primiparous						
No	1 (ref)	1 (ref)	1 (ref)	1 (ref)	1 (ref)	1 (ref)
Yes	2.88 (2.59–3.19)	2.44 (2.19–2.72)	2.58 (2.06–3.24)	1.63 (1.27–2.11)	2.83 (2.51–3.18)	2.42 (2.14–2.74)

^a^Calendar birth year of the woman and the first four principal components were adjusted in the crude and adjusted odds ratio

^b^The variables in the table were mutually adjusted and further adjusted for age and age squared in the adjusted OR

**Fig. 2:**
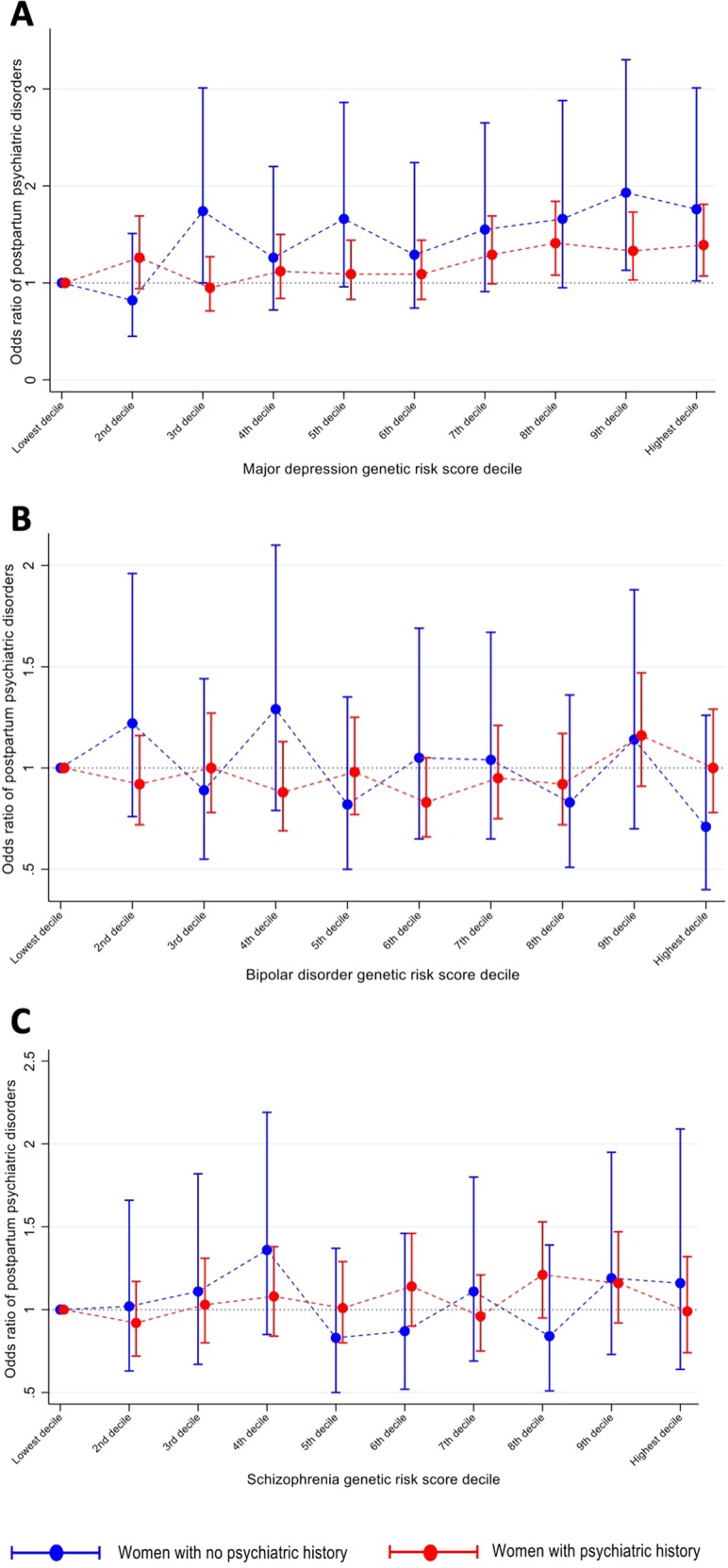
Odds ratio of postpartum psychiatric disorders by genetic risk scores for major depression, bipolar disorder, and schizophrenia. Odds ratio of postpartum psychiatric disorders by **a** major depression, **b** bipolar disorder, and **c** schizophrenia GRS decile with reference to the lowest decile-panel among women with and with no psychiatric history before delivery. All are adjusted for calendar birth year of the woman, first four principal components, parental psychiatric history, parental country of origin, age and age squared at the index delivery, and primiparity

Genetic risk score for MD significantly predicted postpartum psychiatric disorders as well as psychiatric disorders at any time point. A 10-decile increase in MD GRS was associated with an increased risk of postpartum psychiatric disorders (OR: 1.73, 95% CI: 1.46–2.05), in both women with no personal psychiatric history (OR: 1.88, 95% CI: 1.26–2.81) and women with personal psychiatric history (OR: 1.44, 95% CI: 1.19–1.74). Risk of postpartum psychiatric disorders was significantly increased for the highest deciles of MD GRS compared to the lowest decile. (Table 3, Fig. 2a) (Full models in Supplementary Tables S4, S5, S6; Models by decile, Supplementary Table S7). Adjusted pseudo-R^2^ was 0.40% for MD GRS among women with no prior personal psychiatric history and 0.20% among women with previous personal psychiatric history. Area under the covariate-adjusted ROC curve (AUC) was 0.54 (95% CI: 0.52–0.58) for MD GRS to predict postpartum psychiatric disorders among women with no prior personal psychiatric history and 0.52 (95% CI: 0.51–0.54) among women with previous psychiatric history (Fig. S1). Associations between MD GRS and psychiatric disorders at any time (OR 2.48, 95% CI: 2.06–2.99) (Table 3; by decile, Supplementary Table S8) were stronger than associations with postpartum disorders (*P*-value for OR difference was 0.007).

**Table 3:** Odds ratio of postpartum psychiatric disorders and psychiatric disorders at any time by the genetic risk scores and previous psychiatric history

Genetic risk scores	Full cohort				Women with no psychiatric history				Women with psychiatric history				*P* for Interaction
	Crude OR^a^ (95% CI)	*P*	Adjusted OR (95% CI)^a,b^	*P*	Crude OR^a^ (95% CI)	*P*	Adjusted OR (95% CI)^a,b^	*P*	Crude OR^a^ (95% CI)	*P*	Adjusted OR (95% CI)^a,b^	*P*	
*Postpartum psychiatric episodes*													
Genetic risk score for major depression													
Per one-standard-deviation increase	1.20 (1.15–1.26)	<0.001	1.16 (1.10–1.22)	<0.001	1.31 (1.18–1.45)	<0.001	1.18 (1.05–1.31)	0.006	1.13 (1.08–1.19)	<0.001	1.10 (1.04–1.16)	0.001	0.160
Per 10-decile increase	1.93 (1.65–2.27)	<0.001	1.73 (1.46–2.05)	<0.001	2.52 (1.76–3.62)	<0.001	1.88 (1.26–2.81)	0.002	1.57 (1.31–1.89)	<0.001	1.44 (1.19–1.74)	<0.001	0.115
Genetic risk score for bipolar disorder													
Per one-standard-deviation increase	1.03 (0.98–1.08)	0.304	1.02 (0.97–1.08)	0.441	0.95 (0.85–1.07)	0.419	0.94 (0.83–1.06)	0.315	1.03 (0.97–1.08)	0.382	1.03 (0.97–1.09)	0.398	0.138
Per 10-decile increase	1.09 (0.93–1.27)	0.297	1.07 (0.90–1.26)	0.460	0.89 (0.62–1.28)	0.536	0.81 (0.54–1.22)	0.319	1.08 (0.90–1.29)	0.415	1.08 (0.90–1.31)	0.409	0.178
Genetic risk score for schizophrenia													
Per one-standard-deviation increase	1.09 (1.03–1.15)	0.005	1.06 (1.00–1.13)	0.049	1.08 (0.95–1.24)	0.252	1.01 (0.87–1.17)	0.939	1.06 (0.99–1.13)	0.084	1.05 (0.98–1.13)	0.170	0.559
Per 10-decile increase	1.29 (1.09–1.52)	0.002	1.22 (1.02–1.45)	0.029	1.25 (0.85–1.82)	0.255	0.99 (0.66–1.51)	0.980	1.22 (1.01–1.47)	0.039	1.19 (0.97–1.45)	0.092	0.567
*Psychiatric episodes at any time*													
Genetic risk score for major depression													
Per one-standard-deviation increase	1.33 (1.26–1.40)	<0.001	1.30 (1.23–1.37)	<0.001	–	–	–	–	–	–	–	–	–
Per 10-decile increase	2.66 (2.21–3.19)	<0.001	2.48 (2.06–2.99)	<0.001	–	–	–	–	–	–	–	–	–
Genetic risk score for bipolar disorder													
Per one-standard-deviation increase	1.07 (1.01–1.14)	0.016	1.06 (1.00–1.13)	0.045	–	–	–	–	–	–	–	–	–
Per 10-decile increase	1.25 (1.04–1.50)	0.020	1.20 (0.99–1.45)	0.061	–	–	–	–	–	–	–	–	–
Genetic risk score for schizophrenia													
Per one-standard-deviation increase	1.16 (1.08–1.24)	<0.001	1.13 (1.05–1.21)	0.001	–	–	–	–	–	–	–	–	–
Per 10-decile increase	1.47 (1.21–1.79)	<0.001	1.36 (1.12–1.66)	0.002	–	–	–	–	–	–	–	–	-

Adjusted pseudo-R^2^ = 0.15% for genetic risk score of major depression, 0.01% for bipolar disorder and 0.03% for schizophrenia. Pseudo-R^2^ = 0.40% for genetic risk score of major depression, 0.04% for bipolar disorder and 0.00% for schizophrenia among women with no previous psychiatric history. Pseudo-R^2^ = 0.20% for major depression, 0.01% for bipolar disorder and 0.03% for schizophrenia among women with previous psychiatric history

^a^Calendar birth year of the woman and the first 4 principal components were adjusted in the crude and adjusted odds ratio

^b^Further adjusted for parental psychiatric history, parental country of origin, age and age squared at the index delivery, and primiparous

We found no associations between GRS for BD and postpartum psychiatric disorders, either overall or stratified by psychiatric history (Table 3, Fig. 2b). There was a slight association between GRS for SCZ and postpartum psychiatric disorders overall (Table 3, Fig. 2c). Pseudo-R^2^ for BD and SCZ GRS were small (Table 3) and AUC for BD and SCZ GRS to predict postpartum psychiatric disorders was smaller than that of MD GRS (Supplementary Figs. S2, S3). In our analysis of cases of psychiatric disorders at any time, we found GRS for BD and SCZ predicted psychiatric disorders, though less strongly than for GRS MD.

We observed similar trends between GRS and postpartum psychiatric disorders when we generated scores from SNPs with *P* ≤ 1.0 in the GWAS training-set (Supplementary Table S9). We found no significant differences when restricting only to women with parents of Danish origin (Supplementary Table S10).

## Discussion

### Summary of results

In a large sample of women who had given birth, we found that genetic risk score for lifetime MD was associated with postpartum psychiatric episodes in both women with no psychiatric history (OR: 1.88, 95% CI: 1.26–2.81 for a 10-decile increase in GRS) and women with psychiatric history (OR: 1.44, 95% CI: 1.19–1.74 for a 10-decile increase in GRS). In contrast, we found no significant associations between BD risk scores and postpartum psychiatric episodes. We found small associations between SCZ risk scores and postpartum disorders in the cohort unstratified by psychiatric history. Previously identified risk factors for postpartum psychiatric disorders including primiparity and family history were confirmed in our sample.

### Utility of a GRS

Prior studies have shown MD GRS based on the PGC discovery sample to be a strong predictor of major depression in several other target samples^46^ suggesting the utility of MD GRS as a tool to investigate how additional risk factors may influence risk of MD in genetically susceptible groups, as has been done for other disorders such as schizophrenia^48^. As our cases predominantly consist of depression diagnoses and women prescribed antidepressants, it is encouraging that MD GRS predicts psychiatric episodes in our target sample. However, both the pseudo-R^2^ of MD GRS and AUC of MD GRS to predict postpartum psychiatric episodes were small, indicating that MD GRS should not yet be used in a clinical setting to assess risk of postpartum psychiatric episodes. Increasingly large MD discovery samples are necessary to construct accurate predictive GRS, and replication in multiple perinatal populations will be required before GRS can be employed for screening or therapeutic interventions.

Previously identified risk factors including primiparity and family history of psychiatric disorders were confirmed in our sample. Information about these risk factors is easily attained through patient inquiry and medical record review and is currently the best information available for evaluating risk of postpartum psychiatric disorders in a clinical setting. Nonetheless, associations between MD GRS and postpartum psychiatric disorders indicate that the GRS may provide information about genetic liability for psychiatric illness in addition to family history. In particular, the effect of family history remains unchanged with addition of a MD GRS into the model, and there are significant independent associations of MD GRS with postpartum psychiatric disorder case status in both women with and women without prior personal psychiatric history (Supplementary Table S4). In addition, among women with no previous psychiatric history there is an independent association of MD GRS with postpartum psychiatric disorders, even in the absence of an association of parental psychiatric history (Supplementary Table S4), though this may be an artifact of greater statistical power to detect associations with GRS, a continuous variable, than parental psychiatric history, a dichotomous variable.

The one other study applying GRS to postpartum psychiatric disorders compared postpartum depression cases to population controls and separately compared postpartum depression cases to controls who had given birth. They found that BD GRS explained more variance and was more significant for the subset of women who had given birth than in the sample using population controls (*R*^*2*^ *=* 1.64%, *P* = 3.04 × 10^−5^ versus *R*^*2*^ *=* 1.03%, *P* = 1.75 × 10^−4^)^35^. Importantly, adequate sample sizes of genotyped cases of postpartum depression (PPD) for a GWAS will enable construction of a PPD-specific GRS, but in the meantime, our study provides evidence that GRS for lifetime major depression can be applied in a postpartum population. Although still infeasible for direct application in a perinatal mental health clinical setting, genetic risk scores are being used for research in several disease areas to determine whether people in different strata on the genetic risk distribution differ by response to treatment^49^ or likelihood to be appropriately captured by screening guidelines^50^ and are a step toward precision medicine.

### Comparison with previous results

Interestingly, we did not find an expected association with BD GRS. Prior epidemiologic work demonstrates postpartum psychiatric illness may share more features with bipolar disorder than other psychiatric disorders^20–23,39,51^, particularly for postpartum psychosis^7,52–55^. In the study applying genetic risk scores to PPD, Byrne et al. found a stronger genetic relationship between PPD and BD than PPD and MD by applying genetic risk scores for MD and BD^35^. We hypothesize that the Byrne et al. findings were not replicated in our sample for several reasons. First, case and sample selection differed in the studies. Postpartum psychiatric episodes are heterogeneous and the majority of cases in our sample are most similar to major depression; we would expect stronger associations with the MD GRS. The outcome of PPD in Byrne et al. is also based primarily on self-reported symptoms on the Edinburgh Postnatal Depression Scale^56^ collected in community samples (i.e., not enriched for women seeking in- or out-patient treatment). Second, since the Byrne et al. study was conducted, larger MD GWAS sample sizes have identified new genome-wide significant loci, producing a more powerful MD GRS that explains more variance in genetic liability. Thus, the stronger associations with the MD GRS than BD GRS may be due to the improved statistical power of the MD score.

### Unique concerns and characteristics of investigating genetic etiology of postpartum psychiatric disorders

We applied a broad case definition of postpartum psychiatric disorders, comprising a spectrum of psychiatric diagnoses identified by hospital diagnoses and psychotropic prescriptions and occurring within the first year after childbirth. Although more heterogeneous, we applied a broad definition to ensure we were capturing cases that may be missed in hospital registries, examine similarities and differences with other psychiatric disorders, overcome sample size limitations, and provide a comparable definition to other studies.

Prior studies suggest the etiology of psychiatric episodes occurring immediately after childbirth may differ from later psychiatric episodes^3,7,23,51^, and future work will investigate potential underlying genetic differences as sample sizes grow. Although the first year postpartum may not identify a specific etiologic cut-point, it is a commonly used clinical definition of postpartum psychiatric disorders^38^ and enables detection of women that may delay seeking care. Additionally, in our prior work in a similar cohort, we compared associations of family psychiatric history and postpartum psychiatric disorders at three, six, and twelve months after childbirth and found few differences among the time points^39^, indicating it is reasonable to assess genetic and familial predictors of postpartum psychiatric disorders within the first year after childbirth in this setting. Including the entire first year postpartum can also improve statistical power. Although our sample was selected from a large population-based cohort, sample sizes of women who had given birth were still relatively small.

In Denmark, 85–90% of first depression treatment among people aged 15–44 occurs in a primary care setting^57^, and limiting our cases to only hospital-based psychiatric diagnoses will underrepresent individuals susceptible to mental illness, particularly depression. Therefore, our definition of psychiatric disorders included both hospital diagnosed cases and women who used antidepressant medications. This broader definition aligns with the concept of major depression operationalized by the PGC, which was the source of the summary statistics for the GRS scores. To better understand the genetic architecture of depression and take advantage of existing genomic resources, the PGC has adopted the concept of ‘major depression’ to establish case status, which includes self-declared depression and is broader than major depressive disorder meeting DSM criteria obtained by clinical interview^58^. However, we also acknowledge that a hospital-based diagnosis is more specific and antidepressant medications may be prescribed for conditions other than depression. In Denmark, depression is recorded as the indication for 59% of antidepressant prescriptions^57^. Examining differences by case source may provide information about how including psychotropic prescriptions influence results and also genetic homogeneity by severity, so we conducted a sensitivity analysis categorizing postpartum psychiatric disorders into those defined by hospital diagnosis (Supplementary Table S11a) and psychotropic medication use (Supplementary Table S11b). We found no strong evidence for heterogeneity by subset, however, effect sizes were slightly larger for MD GRS among those defined only by psychotropic medication use, further supporting the similarity of our definition with that of major depression used by the PGC.

While most similar to major depression, our broad definition of postpartum psychiatric disorders included a spectrum of heterogeneous diagnoses that may have different underlying etiology. Prior work has shown that these psychiatric disorders are genetically correlated^59^, and GRS for one psychiatric disorder can be informative for predicting another^60^, but we did not have ample statistical power to assess specific diagnoses separately.

There are several hypotheses about the development of psychiatric disorders following childbirth. One is that additional stressors of childbirth and the postpartum period exacerbate an underlying vulnerability of mental illness. Another is that some women are sensitive to hormonal changes occurring near childbirth^61–63^. To probe these hypotheses and consider childbirth as a potential trigger, we investigated associations between genetic vulnerability for psychiatric disorders and postpartum disorders in the overall cohort and then stratified by psychiatric history prior to childbirth. We found little evidence of statistical differences by prior psychiatric history, but we present stratified results because psychiatric history is such a significant risk factor for postpartum psychiatric disorders^6^ and may provide insight about new-onset postpartum disorders. Tests for interaction generally have low power to reject homogeneity^64^ but this is the largest existing study in this area, and trends observed in women with and without prior psychiatric history can provide the opportunity for comparison in future studies.

In the analyses assessing the outcome of postpartum psychiatric disorders in the full cohort and among women with previous psychiatric history, the control groups included women with psychiatric diagnoses prior to childbirth, which is likely to weaken an association between MD GRS and postpartum psychiatric episodes. Even within these groups, we saw a significant association which may provide further evidence of enhanced genetic vulnerability for postpartum psychiatric disorders. Postpartum disorders were our primary outcome of interest, but to determine how much limited power influenced the ability to detect associations for BD and SCZ risk scores, and understand how including women with psychiatric diagnoses as controls may have influenced our results in women with previous psychiatric history, we conducted an additional analysis comparing all women in our cohort with psychiatric disorders at any time (*N* = 7010) with all women without any psychiatric history at any time (i.e., ‘clean controls’) (*N* = 1840). We found that risk scores for BD and SCZ did predict the outcome of lifetime psychiatric disorders in our sample, however, associations were less strong than for MD (Table 3, Supplementary Table S8).

We also found that associations between MD GRS and psychiatric disorders were stronger for psychiatric disorders at any time than in our primary analyses of postpartum disorders. One might expect these associations to be stronger and more precise since the sample is larger, the controls contain no prior psychiatric episodes, and the cases include women with psychiatric disorders before childbirth, which may occur earlier and indicate greater susceptibility to mental illness. Our results provide evidence that the underlying genetic vulnerability of postpartum disorders in our sample is most similar to that of major depression, but we canot not determine if differences in strength of the associations are due to the sample size, sampling framework, or underlying etiologic and genetic differences. Future studies enabling an analysis of a GRS specifically for postpartum psychiatric disorders will help distinguish whether there are unique genetic features of postpartum disorders and potentially identify vulnerable risk groups both within and outside of the postpartum period.

### Strengths and Limitations

This study has several other limitations. The oldest participants in our study are 34 years with a mean age of 25.9 years (SD 3.8), representing a relatively young cohort that potentially limits generalizability to other age groups. Additionally, most of the sample was primiparous, and thus lifetime risk of postpartum psychiatric disorders would not necessarily have been captured by these first births. The iPSYCH case selection influenced the proportion and prevalence of specific psychiatric diagnoses in the parent study^36^, resulting in more cases than controls and a higher proportion of severe psychiatric disorders (e.g., schizophrenia) compared to the general population. Including antidepressant use in the case definition for the present study consequently resulted in cases that were primarily depression cases. Data for prescription use was only available after 1995, so parental psychiatric history was limited only to diagnosed psychiatric disorders, which most likely represent more severe cases.

Despite these limitations, the study has a number of strengths. This is the first study to apply psychiatric GRS in a population only of postpartum women. The study uses objective and well-validated^65–67^ register-based treated psychiatric episodes (i.e., diagnostic codes and redeemed prescriptions) rather than self-reported outcomes. Linkage with national registers also enables assessment of additional risk factors such as prior psychiatric history and family history. Finally, selection from a nationally representative sample increases our generalizability to broader populations, which is especially important when assessing other risk factors in addition to genetic liability.

## Conclusions

We show evidence that genetic liability for major depression is associated with postpartum psychiatric disorders, suggesting genetic risk scores can provide additional information about risk not encompassed solely in simple measures of family history, however, the predictive ability of MD GRS to identify postpartum psychiatric disorders was small. Our findings demonstrate that although GRS is not yet appropriate as a clinical tool, genetic information about the risk of lifetime psychiatric illness can reasonably be applied to the postpartum period in the research setting. As sample sizes of genetic studies in postpartum populations grow, applying GRS to investigate shared genetic mechanisms may provide insight into distinct attributes of postpartum disorders. If replicated, our results could act as first steps in guiding future work focused on investigations that integrate studies in genetic and environmental risk factors to ultimately define vulnerable groups at risk for psychiatric disorders following childbirth.

## Supplementary information

Supplementary Material
