# Supplementary material for: Genetic risk scores for major psychiatric disorders and the risk of postpartum psychiatric disorders
Source: Transl Psychiatry. 2019 Nov 11;9:288. doi: 10.1038/s41398-019-0629-9 (PMC6848186; doi:10.1038/s41398-019-0629-9)
Supplement: Supplementary file 1 — Supplementary Material [file 41398_2019_629_MOESM1_ESM.docx]

**Supplementary Material**

Supplementary Methods

Table S1. Case definition of postpartum psychiatric disorders by previous psychiatric history

Table S2. Characteristics of genetic risk scores

Table S3. Adjusted odds ratios for postpartum psychiatric disorders during the follow-up period according to baseline characteristics (Full model corresponding to Table 2)

Table S4. Odds ratio of postpartum psychiatric disorders by the major depression genetic risk score and previous psychiatric history (Full model for Table 3, major depression)

Table S4a. Per one-standard-deviation increase

Table S4b. Per 10-decile increase

Table S5. Odds ratio of postpartum psychiatric disorders by the bipolar disorder genetic risk score and previous psychiatric history (Full model for Table 3, bipolar disorder)

Table S5a. Per one-standard-deviation increase

Table S5b. Per 10-decile increase

Table S6. Odds ratio of postpartum psychiatric disorders by the schizophrenia genetic risk score and previous psychiatric history (Full model for Table 3, schizophrenia)

Table S6a. Per one-standard-deviation increase

Table S6b. Per 10-decile increase

Table S7. Odds ratio of postpartum psychiatric disorders by the genetic risk scores continuously and measured continuously and in deciles with reference to the lowest decile-panel

Table S8. Sanity check on odds ratio of *any psychiatric disorders* by the genetic risk scores continuously and measured continuously and in deciles with reference to the lowest decile-panel

Table S9. Odds ratio of postpartum psychiatric disorders by the genetic risk scores continuously and measured continuously and in deciles with reference to the lowest decile-panel by previous psychiatric history (with p_t_≤1.0)

Table S10. The odds ratio of postpartum psychiatric disorders by the genetic risk scores and previous psychiatric history among children born to Danish parents

Table S11. Odds ratio of postpartum psychiatric disorders defined by source of case-definition (hospital ICD diagnosis or prescription use) by the genetic risk scores and previous psychiatric history

Table S11a. Defined by hospital contact, regardless of medication use

Table S11b. Defined by psychotropic prescription only

Figure S1. Covariate-adjusted receiver operating characteristic curves for major depression genetic risk score in distinguishing between postpartum psychiatric disorder cases and controls, by personal psychiatric history

Figure S2. Covariate-adjusted receiver operating characteristic curves for bipolar disorder genetic risk score in distinguishing between postpartum psychiatric disorder cases and controls, by personal psychiatric history

Figure S3. Covariate-adjusted receiver operating characteristic curves for schizophrenia genetic risk score in distinguishing between postpartum psychiatric disorder cases and controls, by personal psychiatric history

Supplementary Methods

*Study design*

Data from Danish population-based registers were linked by unique personal identification numbers, which are assigned to all live-born children and new residents in Denmark and enables linkage across all national registers. The Danish Civil Registration System among others holds information on date of birth, emigration, death, and identity of parents.^1^ We conducted a case-control study selected from the iPSYCH2012 sample, which has been described elsewhere.^2^ Briefly, the iPSYCH2012 sample was selected from the Danish Civil Registration System of all singleton births born between May 1st, 1981 and December 31st, 2005 who were alive and resided in Denmark at one year of age and whose mother was known. The full cohort was then linked to the Danish Psychiatric Central Research Register. All subjects within the full cohort who had a diagnosis of SCZ, autism spectrum disorder, attention-deficit/hyperactivity disorder, BD, and affective disorder (including BD) were identified as cases in the iPSYCH2012 sample (N=57 377). A random sample of 30 000 subjects (i.e. the subcohort), was selected from the full cohort from which the cases were drawn (Figure 1).^3^ This random sample represents a population sample to be used as controls in the genetic analysis. However, since these 30 000 subjects were chosen randomly from the full cohort, some also have psychiatric disorders.

*Study population*

For the current study, eligible study subjects were all women born during 1981–1999 and included in the iPSYCH2012 study sample who gave birth to at least one child before December 31st, 2015 and passed genetic quality control to enable calculation of genetic risk scores for MDD, BP, and SCZ (N=8 931) (Figure 1). Hence, from this design women are aged between 18 to 34 years, and the majority are primiparous (64%). We excluded 11 women who emigrated before their first delivery and 70 women that could not be linked to their fathers in the register. Altogether 8,850 women were left in our final analysis.

*Postpartum psychiatric disorders*

We defined a postpartum psychiatric disorder either as (1) a treated psychiatric episode recorded in the Danish Psychiatric Central Research Register or (2) at least one redeemed prescription of psychotropic drugs recorded in the Danish National Prescription Registry within 12 months after delivery. Consequently, our case group consisted of moderate (prescription defined) and severe (psychiatric specialty defined) cases (Table S1). A 12-month risk period was selected because although the Diagnostic and Statistical Manual of Mental Disorders, 5^th^ edition^4^ and the International Classification of Diseases, 10^th^ revision^5^ both define the postpartum period as the first 4-6 weeks after childbirth, the World Health Organization now also considers a broader definition extending to one year.^6^

The Danish Psychiatric Central Research Register contains information on inpatient contacts at psychiatric hospitals and psychiatric wards from 1969 and onwards. From 1995 psychiatric outpatient treatment and emergency room contacts were included .^7^ The International Classification of Diseases, 8^th^ Revision (ICD-8) codes were used to encode the disease diagnosis until 1993 and 10^th^ Revision (ICD-10) from 1994 onward. Mothers in the cohort gave birth after 1997, so ICD-8 codes were only used to define psychiatric history; ICD-10 codes were used to define cases. Treated psychiatric episodes included any psychiatric diagnosis (Primary diagnosis, ICD-10 codes F00–F99), excluding mental retardation and substance abuse (ICD-10 codes F10–F19 and F70–F79). The Danish National Prescription Registry was established in 1995, and contains information on all prescriptions dispensed at community pharmacies in Denmark.^8^ The register includes data on drug class stored as Anatomical Therapeutic Chemical (ATC) classification system and the dispensing date. We included psychotropic drugs of ATC codes N05 (psycholeptics) and N06 (psychoanaleptics). To examine the distribution of subtypes of disorders in our sample, we further categorized cases of psychiatric disorders as diagnosed (1) SCZ and related disorders (ICD-10 codes F20–F29), (2) bipolar disorder (ICD-10 codes F30–F31), (3) unipolar depression(ICD-10 codes F32–F33), (4) neurotic, stress-related, and somatoform disorder (ICD-10 codes F40–F48), or (5) other psychiatric diagnosis; or a dispensed prescription of (1) antidepressant medication (ATC codes N06A), or (2) other psychotropic medication (ATC codes N05–N06 excluding N06A). In the case that a woman had multiple diagnoses, the first diagnosis in the postpartum period was used to determine the subtype category.

*Psychiatric history*

We defined previous psychiatric history as one in-patient or out-patient treatment for psychiatric disorders (ICD-8 codes 290–309 excluding 303 and 304; ICD-10 codes F00–F99 excluding F10–F19 and F70–F79) or one redeemed prescription for psychotropic medications before the date of delivery. We similarly defined parental psychiatric history as one in-patient or out-patient treatment for psychiatric disorders in a mother or father before the date of delivery.

*Genotyping, quality control, and imputation*

The Danish Newborn Screening Biobank stored dried blood spots taken at birth from nearly all infants born in Denmark since May 1^st^ 1981.^9^ Genetic data were extracted from the dried blood spot samples stored in this biobank, whole-genome amplified (in triplicate using the Qiagen REPLI-g mini kit and the 3 separate reactions were pooled), and genotyped with Illumina Infinium HD Human610-Quad BeadChip.^10^ Quality control and imputation were conducted using the Ricopili pipeline (https://sites.google.com/a/broadinstitute.org/ricopili/).^11^ The default parameters for retention of single nucleotide polymorphisms (SNPs) and subjects are: SNP missingness < 0.05 (before sample removal); sample missingness < 0.02; autosomal heterozygousity deviation F within +/-0.2 in cases in controls; SNP missingness < 0.02 (after sample removal); difference in SNP missingness between cases and controls < 0.02; SNP Hardy-Weinberg equilibrium (*P* > 10^-6^ in controls or *P* > 10^-10^ in cases). Sex violations in which genetic sex did not match pedigree sex were excluded.

Genotype imputation was performed using a stepwise approach in IMPUTE2/SHAPEIT with default parameters. The reference set consisted of phased haplotypes from the 1000 Genomes Project (phase 3). SNPs with high imputation quality (INFO score > 0.8) and low missingness < 0.01 were retained for analysis. SNPs were LD pruned (r^2^ > 0.02) and clumped (within 500 kb) to select independent SNPs and uncommon SNPs were excluded (MAF < 0.01). Principal components to be used as covariates were created from the post-imputation dataset in EIGENSOFT and ancestry outliers were excluded. Identical samples and closely related individuals were also excluded.

*Genetic risk scores*

We computed genetic risk scores for each individual using PLINK v1.9, calculated as the sum of the allele dosages weighted by the natural log-transformed odds ratio of the risk allele. We generated scores for MDD,^12^ BD,^13^ and SCZ^14^ based on genome-wide data from Psychiatric Genomics Consortium (PGC) discovery sample including 23andMe. Individuals in the iPSYCH2012 sample were excluded. We selected SNPs associated with MDD, BP, and SCZ at a p-value threshold of 0.05 or lower, which were reported as maximising out-of-sample prediction into multiple cohorts of the study specific disorders (Table S2, in the Supplement).^14–16^

*Statistical analysis*

Statistical analyses were performed using the statistical software package Stata 13.1. Binary logistic regression models were used to estimate the odds ratios (ORs) of postpartum psychiatric disorders with 95% confidence intervals (CIs). We converted genetic risk scores into z-scores according to the means and standard deviations from the distributions in women born during 1981–1999 from the subcohort (i.e., the random sample from the whole Danish population) based on the following formula: (observed value -mean)/standard deviation. First, we included the standardized genetic risk scores as continuous variables. We also divided the standardized genetic risk scores into deciles according to the distribution and included them in the models as dummy variables with the lowest decile as the reference group. We adjusted for parental psychiatric history before the index delivery (yes/no), parental country of origin (Denmark/at least one parent outside Denmark), primiparity (yes/no), and age at the index delivery (age+ age squared). To account for ancestry differences, we adjusted for the first 4 principal components estimated from genome-wide SNP genotypes.^17^ To address the possible secular trends in diagnostic practices and to account for the fact that fewer blood spots were retrievable among women born in the earlier years, we included woman's calendar year of birth (1981–1985, 1986–1990, or 1991–1999) as a covariate. To test whether the associations between genetic risk scores and postpartum psychiatric disorders were modified by previous psychiatric history, all analyses were stratified by previous psychiatric history before delivery.

To determine whether results were influenced by the p-value threshold, we repeated our analysis including all GWAS SNPs for MDD, BP, and SCZ. Our primary outcome of interest was a psychiatric disorder in the postpartum period, but to test to which extent genetic risk scores in general predict psychiatric disorders in our sample, we also conducted an analysis in which cases consisted of psychiatric disorders at any time point and controls were individuals free of psychiatric disorders at any time.

**Table S1.**Case definition of postpartum psychiatric disorders according to previous psychiatric history (n=3 021)

|  | **Hospital contact only** | **Prescription of psychotropic medication only** | **Both hospital contact  + prescription of psychotropic medication** |
| --- | --- | --- | --- |
| **Cases with no previous psychiatric history (n=479)** | 51 (10.6%) | 186 (38.8%) | 242 (50.5%) |
| **Cases with previous psychiatric history**  **(n=2 542)** | 222 (8.7%) | 1,547 (60.9%) | 773 (30.4%) |
| **All postpartum psychiatric cases**  **(n=3 021)** | 273 (9.0%) | 1,733 (57.4%) | 1,015 (33.6%) |

**Table S2.** Characteristics of genetic risk scores

|  |  | **Number of SNPs included in each score** | | |
| --- | --- | --- | --- | --- |
| **Genetic risk score** | **P-value threshold from the discovery dataset** | **MDD** | **BD** | **SCZ** |
| S1 | Pt < 0.00000005 | 36 | 17 | 109 |
| S2 | Pt < 0.000001 | 101 | 64 | 243 |
| S3 | Pt < 0.0001 | 710 | 468 | 1 288 |
| S4 | Pt < 0.001 | 2 608 | 1 750 | 3 430 |
| S5 | Pt < 0.01 | 10 549 | 7 100 | 10 622 |
| S6 | Pt < 0.05 | 28 861 | 19 265 | 24 755 |
| S7 | Pt < 0.1 | 44 721 | 29 587 | 35 792 |
| S8 | Pt < 0.2 | 68 474 | 44 658 | 51 892 |
| S9 | Pt < 0.5 | 115 408 | 72 386 | 81 340 |
| S10 | Pt < 1.00 | 151 762 | 90 608 | 102 366 |

# **Table S3.** Adjusted odds ratios for postpartum psychiatric disorders during the follow-up period according to baseline characteristics (Full model corresponding to Table 2)^1^

| **Characteristics** | Women with no previous psychiatric history | | | Women with previous psychiatric history | | |
| --- | --- | --- | --- | --- | --- | --- |
|  | OR (95% CI) | SE | p-value | OR (95% CI) | SE | p-value |
| **Parental psychiatric history** |  |  |  |  |  |  |
| No | 1 (ref) |  |  | 1 (ref) |  |  |
| Yes | 1.08 (0.81 – 1.43) | 0.155 | 0.599 | 1.14 (1.02 – 1.28) | 0.068 | 0.027 |
| **Parental country of origin** |  |  |  |  |  |  |
| Denmark | 1 (ref) |  |  | 1 (ref) |  |  |
| At least one parent outside Denmark | 1.43 (0.86 – 2.36) | 0.366 | 0.165 | 0.95 (0.76 – 1.20) | 0.112 | 0.695 |
| **Primiparous** |  |  |  |  |  |  |
| No | 1 (ref) |  |  | 1 (ref) |  |  |
| Yes | 1.63 (1.27 – 2.11) | 0.211 | <0.001 | 2.42 (2.14 – 2.74) | 0.152 | <0.001 |
| **Age** | 3.32 (2.23 – 4.93) | 0.672 | <0.001 | 1.15 (0.94 – 1.41) | 0.119 | 0.176 |
| **Age squared** | 0.97 (0.96 – 0.98) | 0.004 | <0.001 | 0.99 (0.99 – 1.00) | 0.002 | 0.001 |
| **First PC** | 2.19×10^-39^ (1.50×10^-70^ – 3.22×10^-8^) | 8.03×10^-38^ | 0.015 | 1.16×10^-6^ (8.44×10^-18^ – 1.59×10^5^) | 1.5×10^-5^ | 0.296 |
| **Second PC** | 1.77×10^-22^ (4.01×10^-50^ – 7.86×10^5^) | 5.76×10^-21^ | 0.123 | 3.30×10^-7^ (1.99×10^-16^ – 5.49×10^2^) | 3.58×10^-6^ | 0.168 |
| **Third PC** | 2.73×10^-11^ (3.31×10^-30^ – 2.25×10^8^) | 6.07×10^-10^ | 0.274 | 2.06×10^3^ (9.42×10^-6^ – 4.51×10^11^) | 2.02×10^4^ | 0.436 |
| **Fourth PC** | 6.87×10^-8^ (2.01×10^-25^ – 2.35×10^10^) | 1.42×10^-6^ | 0.423 | 3.55×10^2^ (1.43×10^-6^ – 8.79×10^10^) | 3.50×10^3^ | 0.552 |
| **Calendar birth year of the woman** |  |  |  |  |  |  |
| 1981–1985 | 1 (ref) |  |  | 1 (ref) |  |  |
| 1986–1990 | 0.38 (0.30 – 0.49) | 0.048 | <0.001 | 0.35 (0.30 – 0.40) | 0.024 | <0.001 |
| 1991–1999 | 0.16 (0.10 – 0.26) | 0.399 | <0.001 | 0.12 (0.09 – 0.15) | 0.013 | <0.001 |

^1^ The variables in the table were mutually adjusted.

# **Table S4.** Odds ratio of postpartum psychiatric disorders by the major depression genetic risk score and previous psychiatric history (Full model for Table 3, major depression)

**Table S4a**. Full models for Table 3, major depression; Per one-standard deviation increase

|  | Women with no previous psychiatric history | | | Women with previous psychiatric history | | |
| --- | --- | --- | --- | --- | --- | --- |
|  | OR (95% CI) | SE | p-value | OR (95% CI) | SE | p-value |
| **GRS for major depression**  **Per 1-SD increase** | 1.18 (1.05 – 1.32) | 0.070 | 0.006 | 1.10 (1.04 – 1.16) | 0.031 | 0.001 |
| **Parental psychiatric history** |  |  |  |  |  |  |
| No | 1 (ref) |  |  | 1 (ref) |  |  |
| Yes | 1.07 (0.80 – 1.41) | 0.154 | 0.660 | 1.14 (1.01 – 1.28) | 0.067 | 0.033 |
| **Parental country of origin** |  |  |  |  |  |  |
| Denmark | 1 (ref) |  |  | 1 (ref) |  |  |
| At least one parent outside Denmark | 1.44 (0.87 – 2.38) | 0.370 | 0.154 | 0.94 (0.75 – 1.19) | 0.111 | 0.629 |
| **Primiparous** |  |  |  |  |  |  |
| No | 1 (ref) |  |  | 1 (ref) |  |  |
| Yes | 1.65 (1.28 – 2.12) | 0.214 | <0.001 | 2.42 (2.14 – 2.74) | 0.151 | <0.001 |
| **Age** | 3.32 (2.23 – 4.94) | 0.674 | <0.001 | 1.15 (0.94 – 1.41) | 0.119 | 0.176 |
| **Age squared** | 0.97 (0.96 – 0.98) | 0.004 | <0.001 | 0.99 (0.99 – 1.00) | 0.002 | 0.001 |
| **First PC** | 2.79×10^-43^ (3.17×10^-74^ – 2.45×10^-12^) | 1.01×10^-41^ | 0.007 | 2.83×10^-8^ (1.86×10^-19^ – 4.30×10^3^) | 3.71×10^-7^ | 0.186 |
| **Second PC** | 4.05×10^-20^ (7.80×10^-48^ – 2.10×10^8^) | 1.32×10^-18^ | 0.170 | 1.22×10^-5^ (6.68×10^-15^ – 2.23×10^4^) | 3.27×10^-4^ | 0.298 |
| **Third PC** | 1.29×10^-11^ (2.53×10^-30^ – 6.58×10^7^) | 2.83×10^-10^ | 0.254 | 4.20×10^2^ (1.87×10^-6^ – 9.45×10^10^) | 4.12×10^3^ | 0.538 |
| **Fourth PC** | 1.53×10^-7^ (4.03×10^-25^ – 5.80×10^10^) | 3.16×10^-6^ | 0.447 | 5.47×10^2^ (2.24×10^-6^ – 1.34×10^11^) | 5.39×10^3^ | 0.522 |
| **Calendar birth year of the woman** |  |  |  |  |  |  |
| 1981–1985 | 1 (ref) |  |  | 1 (ref) |  |  |
| 1986–1990 | 0.39 (0.30 – 0.50) | 0.049 | <0.001 | 0.35 (0.30 – 0.40) | 0.024 | <0.001 |
| 1991–1999 | 0.17 (0.10 – 0.27) | 0.049 | <0.001 | 0.12 (0.09 – 0.15) | 0.013 | <0.001 |

**Table S4b.** Full model for Table 3, major depression; Per 10-decile increase

| **Characteristics** | Women with no previous psychiatric history | | | Women with previous psychiatric history | | |
| --- | --- | --- | --- | --- | --- | --- |
|  | OR (95% CI) | SE | p-value | OR (95% CI) | SE | p-value |
| **GRS for major depression**  **Per 10-decile increase** | 1.88 (1.26 – 2.81) | 0.385 | 0.002 | 1.43 (1.19 – 1.74) | 0.140 | <0.001 |
| **Parental psychiatric history** |  |  |  |  |  |  |
| No | 1 (ref) |  |  | 1 (ref) |  |  |
| Yes | 1.06 (0.80 – 1.41) | 0.153 | 0.669 | 1.14 (1.01 – 1.28) | 0.068 | 0.033 |
| **Parental country of origin** |  |  |  |  |  |  |
| Denmark | 1 (ref) |  |  | 1 (ref) |  |  |
| At least one parent outside Denmark | 1.45 (0.88 – 2.40) | 0.372 | 0.145 | 0.94 (0.75 – 1.19) | 0.111 | 0.619 |
| **Primiparous** |  |  |  |  |  |  |
| No | 1 (ref) |  |  | 1 (ref) |  |  |
| Yes | 1.70 (0.96 – 0.98) | 0.215 | <0.001 | 2.42 (2.14 – 2.74) | 0.152 | <0.001 |
| **Age** | 3.33 (2.24 – 4.96) | 0.676 | <0.001 | 1.15 (0.94 – 1.41) | 0.119 | 0.178 |
| **Age squared** | 0.97 (0.96 – 0.98) | 0.004 | <0.001 | 0.99 (0.99 – 1.00) | 0.002 | 0.001 |
| **First PC** | 1.86×10^-42^ (8.39×10^-74^ – 4.14×10^-11^) | 6.86×10^-41^ | 0.009 | 1.31×10^-7^ (9.77×10^-19^ – 1.76×10^4^) | 1.71×10^-6^ | 0.225 |
| **Second PC** | 9.38×10^-21^ (1.08×10^-48^ – 8.17×10^7^) | 3.08×10^-19^ | 0.160 | 3.73×10^-6^ (2.28×10^-15^ – 6.09×10^3^) | 4.01×10^-5^ | 0.248 |
| **Third PC** | 7.10×10^-11^ (7.40×10^-30^ – 6.81×10^8^) | 1.58×10^-9^ | 0.295 | 9.56×10^2^ (4.41×10^-6^ – 2.07×10^11^) | 9.36×10^3^ | 0.483 |
| **Fourth PC** | 1.44×10^-7^ (3.67×10^-25^ – 5.65×10^10^) | 2.98×10^-6^ | 0.446 | 3.36×10^2^ (1.42×10^-6^ – 7.98×10^10^) | 3.31×10^3^ | 0.554 |
| **Calendar birth year of the woman** |  |  |  |  |  |  |
| 1981–1985 | 1 (ref) |  |  | 1 (ref) |  |  |
| 1986–1990 | 0.38 (0.30 – 0.49) | 0.048 | <0.001 | 0.35 (0.30 – 0.40) | 0.024 | <0.001 |
| 1991–1999 | 0.17 (0.10 – 0.27) | 0.041 | <0.001 | 0.12 (0.09 – 0.15) | 0.013 | <0.001 |

# **Table S5.** Odds ratio of postpartum psychiatric disorders by the bipolar genetic risk score and previous psychiatric history (Full model for Table 3, bipolar disorder)

**Table S5a.** Full model for Table 3, bipolar disorder; Per one-standard deviation increase

| **Characteristics** | Women with no previous psychiatric history | | | Women with previous psychiatric history | | |
| --- | --- | --- | --- | --- | --- | --- |
|  | OR (95% CI) | SE | p-value | OR (95% CI) | SE | p-value |
| **GRS for bipolar disorder**  **Per 1-SD increase** | 0.94 (0.83 – 1.06) | 0.060 | 0.315 | 1.03 (0.97 – 1.09) | 0.031 | 0.398 |
| **Parental psychiatric history** |  |  |  |  |  |  |
| No | 1 (ref) |  |  | 1 (ref) |  |  |
| Yes | 1.08 (0.81 – 1.43) | 0.155 | 0.593 | 1.14 (1.01 – 1.28) | 0.068 | 0.028 |
| **Parental country of origin** |  |  |  |  |  |  |
| Denmark | 1 (ref) |  |  | 1 (ref) |  |  |
| At least one parent outside Denmark | 1.45 (0.88 – 2.40) | 0.372 | 0.147 | 0.95 (0.75 – 1.20) | 0.112 | 0.664 |
| **Primiparous** |  |  |  |  |  |  |
| No | 1 (ref) |  |  | 1 (ref) |  |  |
| Yes | 1.64 (1.28 – 2.12) | 0.213 | <0.001 | 2.42 (2.14 – 2.74) | 0.152 | <0.001 |
| **Age** | 3.31 (2.22 – 4.92) | 0.670 | <0.001 | 1.15 (0.94 – 1.41) | 0.119 | 0.170 |
| **Age squared** | 0.97 (0.96 – 0.98) | 0.004 | <0.001 | 0.99 (0.99 – 1.00) | 0.002 | 0.001 |
| **First PC** | 4.01×10^-36^ (8.45×10^-68^ – 1.91×10^-4^) | 1.49×10^-34^ | 0.029 | 7.16×10^-8^ (2.35×10^-19^ – 2.19×10^4^) | 9.66×10^-7^ | 0.223 |
| **Second PC** | 2.02×10^-23^ (4.85×10^-51^ – 8.40×10^4^) | 6.55×10^-22^ | 0.107 | 5.97×10^-7^ (3.42×10^-16^ – 1.04×10^3^) | 6.48×10^-6^ | 0.187 |
| **Third PC** | 6.11×10^-11^ (8.11×10^-30^ – 4.60×10^8^) | 1.36×10^-9^ | 0.289 | 2.20×10^3^ (1.00×10^-5^ – 4.81×10^11^) | 2.15×10^4^ | 0.432 |
| **Fourth PC** | 9.41×10^-8^ (2.68×10^-25^ – 3.30×10^10^) | 1.94×10^-6^ | 0.432 | 2.99×10^2^ (1.19×10^-6^ – 7.53×10^10^) | 2.95×10^3^ | 0.563 |
| **Calendar birth year of the woman** |  |  |  |  |  |  |
| 1981–1985 | 1 (ref) |  |  | 1 (ref) |  |  |
| 1986–1990 | 0.38 (0.30 – 0.49) | 0.048 | <0.001 | 0.35 (0.30 – 0.40) | 0.024 | <0.001 |
| 1991–1999 | 0.17 (0.10 – 027) | 0.040 | <0.001 | 0.12 (0.09 – 0.15) | 0.013 | <0.001 |

**Table S5b**. Full model for Table 3, bipolar disorder; Per 10-decile increase

| **Characteristics** | Women with no previous psychiatric history | | | Women with previous psychiatric history | | |
| --- | --- | --- | --- | --- | --- | --- |
|  | OR (95% CI) | SE | p-value | OR (95% CI) | SE | p-value |
| **GRS bipolar disorder**  **Per 10-decile increase** | 0.81 (0.54 – 1.22) | 0.169 | 0.319 | 1.08 (0.90 – 1.31) | 0.105 | 0.409 |
| **Parental psychiatric history** |  |  |  |  |  |  |
| No | 1 (ref) |  |  | 1 (ref) |  |  |
| Yes | 1.08 (0.81 – 1.43) | 0.156 | 0.593 | 1.14 (1.01 – 1.28) | 0.068 | 0.028 |
| **Parental country of origin** |  |  |  |  |  |  |
| Denmark | 1 (ref) |  |  | 1 (ref) |  |  |
| At least one parent outside Denmark | 1.45 (0.88 – 2.40) | 0.372 | 0.147 | 0.95 (0.75 – 1.20) | 0.112 | 0.664 |
| **Primiparous** |  |  |  |  |  |  |
| No | 1 (ref) |  |  | 1 (ref) |  |  |
| Yes | 1.65 (1.28 – 2.12) | 0.214 | <0.001 | 2.42 (2.14 – 2.74) | 0.152 | <0.001 |
| **Age** | 3.32 (2.23 – 4.93) | 0.671 | <0.001 | 1.15 (0.94 – 1.41) | 0.119 | 0.171 |
| **Age squared** | 0.97 (0.96 – 0.98) | 0.004 | <0.001 | 0.99 (0.99 – 1.00) | 0.002 | 0.001 |
| **First PC** | 2.90×10^-37^ (2.06×10^-68^ – 4.08×10^-6^) | 1.06×10^-35^ | 0.021 | 2.28×10^-7^ (1.24×10^-18^ – 4.17×10^4^) | 3.01×10^-6^ | 0.248 |
| **Second PC** | 7.40×10^-23^ (2.26×10^-50^ – 2.42×10^5^) | 2.39×10^-21^ | 0.115 | 3.25×10^-7^ (1.95×10^-16^ – 5.42×10^2^) | 3.52×10^-6^ | 0.168 |
| **Third PC** | 3.22×10^-11^ (5.13×10^-30^ – 2.02×10^8^) | 7.10×10^-10^ | 0.274 | 2.91×10^3^ (1.31×10^-5^ – 6.47×10^11^) | 2.85×10^4^ | 0.416 |
| **Fourth PC** | 8.06×10^-8^ (2.40×10^-25^ – 2.70×10^10^) | 1.66×10^-6^ | 0.428 | 2.88×10^2^ (1.14×10^-6^ – 7.26×10^10^) | 2.84×10^3^ | 0.566 |
| **Calendar birth year of the woman** |  |  |  |  |  |  |
| 1981–1985 | 1 (ref) |  |  | 1 (ref) |  |  |
| 1986–1990 | 0.38 (0.30 – 0.49) | 0.048 | <0.001 | 0.35 (0.30 – 0.40) | 0.024 | <0.001 |
| 1991–1999 | 0.17 (0.10 – 0.27) | 0.040 | <0.001 | 0.12 (0.09 – 0.15) | 0.013 | <0.001 |

# **Table S6.** Odds ratio of postpartum psychiatric disorders by the schizophrenia genetic risk scores and previous psychiatric history (Full model for Table 3, schizophrenia)

**Table S6a.** Full models for Table 3, schizophrenia; Per one-standard deviation increase

| **Characteristics** | Women with no previous psychiatric history | | | Women with previous psychiatric history | | |
| --- | --- | --- | --- | --- | --- | --- |
|  | OR (95% CI) | SE | p-value | OR (95% CI) | SE | p-value |
| **GRS for schizophrenia**  **Per 1-SD increase** | 1.00 (0.87 – 1.17) | 0.076 | 0.939 | 1.05 (0.98 – 1.13) | 0.038 | 0.170 |
| **Parental psychiatric history** |  |  |  |  |  |  |
| No | 1 (ref) |  |  | 1 (ref) |  |  |
| Yes | 1.08 (0.81 – 1.43) | 0.155 | 0.601 | 1.14 (1.01 – 1.28) | 0.068 | 0.031 |
| **Parental country of origin** |  |  |  |  |  |  |
| Denmark | 1 (ref) |  |  | 1 (ref) |  |  |
| At least one parent outside Denmark | 1.42 (0.86 – 2.36) | 0.366 | 0.169 | 0.94 (0.75 – 1.19) | 0.111 | 0.616 |
| **Primiparous** |  |  |  |  |  |  |
| No | 1 (ref) |  |  | 1 (ref) |  |  |
| Yes | 1.63 (1.27 – 2.10) | 0.211 | <0.001 | 2.42 (2.14 – 2.74) | 0.151 | <0.001 |
| **Age** | 3.32 (2.23 – 4.93) | 0.671 | <0.001 | 1.15 (0.94 – 1.41) | 0.119 | 0.169 |
| **Age squared** | 0.97 (0.96 – 0.98) | 0.04 | <0.001 | 0.99 (0.99 – 1.00) | 0.002 | 0.001 |
| **First PC** | 9.86×10^-40^ (4.06×10^-72^ – 2.40×10^-7^) | 3.75×10^-38^ | 0.018 | 8.01×10^-10^ (7.59×10^-22^ – 8.44×10^2^) | 1.13×10^-8^ | 0.138 |
| **Second PC** | 1.65×10^-22^ (3.71×10^-50^ – 7.35×10^5^) | 5.37×10^-21^ | 0.123 | 1.64×10^-7^ (9.62×10^-17^ – 2.79×10^2^) | 1.78×10^-6^ | 0.150 |
| **Third PC** | 2.80×10^-11^ (3.42×10^-30^ – 2.30×10^8^) | 6.23×10^-10^ | 0.274 | 3.32×10^3^ (1.50×10^-5^ – 7.32×10^11^) | 3.25×10^4^ | 0.408 |
| **Fourth PC** | 7.92×10^-8^ (1.97×10^-25^ – 3.18×10^10^) | 1.64×10^-6^ | 0.429 | 4.68×10^2^ (1.87×10^-6^ – 1.17×10^11^) | 4.62×10^3^ | 0.533 |
| **Calendar birth year of the woman** |  |  |  |  |  |  |
| 1981–1985 | 1 (ref) |  |  | 1 (ref) |  |  |
| 1986–1990 | 0.38 (0.30 – 0.49) | 0.048 | <0.001 | 0.35 (0.30 – 0.40) | 0.024 | <0.001 |
| 1991–1999 | 0.16 (0.10 – 0.26) | 0.040 | <0.001 | 0.12 (0.09 – 0.15) | 0.013 | <0.001 |

**Table S6b.** Full model for Table 3, schizophrenia; Per 10-decile increase

| **Characteristics** | Women with no previous psychiatric history | | | Women with previous psychiatric history | | |
| --- | --- | --- | --- | --- | --- | --- |
|  | OR (95% CI) | SE | p-value | OR (95% CI) | SE | p-value |
| **GRS for schizophrenia**  **Per 10-decile increase** | 0.99 (0.66 – 1.50) | 0.211 | 0.980 | 1.19 (0.97 – 1.45) | 0.120 | 0.092 |
| **Parental psychiatric history** |  |  |  |  |  |  |
| No | 1 (ref) |  |  | 1 (ref) |  |  |
| Yes | 1.08 (0.81 – 1.43) | 0.156 | 0.598 | 1.14 (1.01 – 1.28) | 0.068 | 0.031 |
| **Parental country of origin** |  |  |  |  |  |  |
| Denmark | 1 (ref) |  |  | 1 (ref) |  |  |
| At least one parent outside Denmark | 1.43 (0.86 – 2.36) | 0.367 | 0.166 | 0.94 (0.74 – 1.18) | 0.111 | 0.592 |
| **Primiparous** |  |  |  |  |  |  |
| No | 1 (ref) |  |  | 1 (ref) |  |  |
| Yes | 1.63 (1.27 – 2.11) | 0.212 | <0.001 | 2.42 (2.14 – 2.74) | 0.152 | <0.001 |
| **Age** | 3.12 (2.23 – 4.93) | 0.672 | <0.001 | 1.15 (0.94 – 1.41) | 0.120 | 0.167 |
| **Age squared** | 0.97 (0.96 – 0.98) | 0.004 | <0.001 | 0.99 (0.99 – 1.00) | 0.002 | 0.001 |
| **First PC** | 2.52×10^-39^ (7.76×10^-71^ – 8.19×10^-8^) | 9.33×10^-38^ | 0.016 | 2.23×10^-8^ (1.06×10^-19^ – 4.69×10^3^) | 2.97×10^-7^ | 0.185 |
| **Second PC** | 1.80×10^-22^ (4.04×10^-50^ – 8.03×10^5^) | 5.85×10^-21^ | 0.123 | 1.36×10^-7^ (7.71×10^-17^ – 2.41×10^2^) | 1.48×10^-6^ | 0.146 |
| **Third PC** | 2.68×10^-11^ (3.17×10^-30^ – 2.26×10^8^) | 5.95×10^-10^ | 0.274 | 5.36×10^3^ (2.36×10^-5^ – 1.21×10^12^) | 5.26×10^4^ | 0.382 |
| **Fourth PC** | 6.76×10^-8^ (1.93×10^-25^ – 2.36×10^10^) | 1.39×10^-6^ | 0.423 | 3.22×10^2^ (1.26×10^-6^ – 8.24×10^10^) | 3.18×10^3^ | 0.559 |
| **Calendar birth year of the woman** |  |  |  |  |  |  |
| 1981–1985 | 1 (ref) |  |  | 1 (ref) |  |  |
| 1986–1990 | 0.38 (0.30 – 0.49) | 0.048 | <0.001 | 0.35 (0.30 – 0.40) | 0.024 | <0.001 |
| 1991–1999 | 0.16 (0.10 – 0.26) | 0.040 | <0.001 | 0.12 (0.09 – 0.15) | 0.013 | <0.001 |

**Table S7.** Odds ratio of postpartum psychiatric disorders by the genetic risk scores measured continuously and in deciles with reference to the lowest decile-panel, by previous psychiatric history

| **Genetic risk scores** | **Women with no psychiatric history** | | | | **Women with psychiatric history** | | | |
| --- | --- | --- | --- | --- | --- | --- | --- | --- |
|  | **Crude OR**  **(95% CI)^1^** | **p-value** | **Adjusted OR (95% CI)^1,2^** | **p-value** | **Crude OR**  **(95% CI)^1^** | **p-value** | **Adjusted OR (95% CI)^1,2^** | **p-value** |
| **Genetic risk score for major depression** |  |  |  |  |  |  |  |  |
| Lowest decile | 1 (ref) |  | 1 (ref) |  | 1 (ref) |  | 1 (ref) |  |
| 2nd decile | 0.96 (0.55–1.67) | 0.876 | 0.82 (0.45–1.51) | 0.525 | 1.30 (0.99–1.71) | 0.060 | 1.26 (0.94–1.69) | 0.119 |
| 3rd decile | 2.13 (1.29–3.51) | 0.003 | 1.74 (1.00–3.01) | 0.049 | 0.95 (0.72–1.25) | 0.710 | 0.95 (0.71–1.27) | 0.719 |
| 4th decile | 1.49 (0.89–2.49) | 0.127 | 1.26 (0.72–2.20) | 0.416 | 1.18 (0.90–1.55) | 0.223 | 1.12 (0.84–1.50) | 0.423 |
| 5th decile | 2.19 (1.33–3.60) | 0.002 | 1.66 (0.96–2.86) | 0.068 | 1.19 (0.92–1.55) | 0.184 | 1.09 (0.83–1.44) | 0.529 |
| 6th decile | 1.63 (0.98–2.71) | 0.059 | 1.29 (0.74–2.24) | 0.374 | 1.13 (0.87–1.47) | 0.363 | 1.09 (0.83–1.44) | 0.534 |
| 7th decile | 2.09 (1.28–3.40) | 0.003 | 1.55 (0.91–2.65) | 0.109 | 1.33 (1.03–1.71) | 0.030 | 1.29 (0.99–1.69) | 0.061 |
| 8th decile | 2.02 (1.22–3.35) | 0.006 | 1.66 (0.95–2.88) | 0.073 | 1.54 (1.20–1.98) | 0.001 | 1.41 (1.08–1.84) | 0.011 |
| 9th decile | 2.43 (1.49–3.96) | <0.001 | 1.93 (1.13–3.30) | 0.016 | 1.47 (1.15–1.88) | 0.002 | 1.33 (1.03–1.73) | 0.031 |
| Highest decile | 2.76 (1.69–4.50) | <0.001 | 1.76 (1.02–3.01) | 0.041 | 1.51 (1.18–1.94) | 0.001 | 1.39 (1.07–1.81) | 0.014 |
| Per one-standard-deviation increase | 1.31 (1.18–1.45) | <0.001 | 1.18 (1.05–1.31) | 0.006 | 1.13 (1.08–1.19) | <0.001 | 1.10 (1.04–1.16) | 0.001 |
| Per 10-decile increase | 2.52 (1.76–3.62) | <0.001 | 1.88 (1.26–2.81) | 0.002 | 1.57 (1.31–1.89) | <0.001 | 1.44 (1.19–1.74) | <0.001 |
| **Genetic risk score for bipolar disorder** |  |  |  |  |  |  |  |  |
| Lowest decile | 1 (ref) |  | 1 (ref) |  | 1 (ref) |  | 1 (ref) |  |
| 2nd decile | 1.02 (0.67–1.56) | 0.931 | 1.22 (0.76–1.96) | 0.410 | 0.95 (0.77–1.19) | 0.682 | 0.92 (0.72–1.16) | 0.468 |
| 3rd decile | 0.81 (0.52–1.26) | 0.347 | 0.89 (0.55–1.44) | 0.629 | 1.00 (0.80–1.26) | 0.978 | 1.00 (0.78–1.27) | 0.981 |
| 4th decile | 1.15 (0.74–1.77) | 0.543 | 1.29 (0.79–2.10) | 0.314 | 0.92 (0.73–1.16) | 0.483 | 0.88 (0.69–1.13) | 0.326 |
| 5th decile | 0.80 (0.51–1.26) | 0.343 | 0.82 (0.50–1.35) | 0.437 | 1.04 (0.83–1.31) | 0.736 | 0.98 (0.77–1.25) | 0.890 |
| 6th decile | 1.06 (0.69–1.64) | 0.775 | 1.05 (0.65–1.69) | 0.844 | 0.87 (0.70–1.09) | 0.221 | 0.83 (0.66–1.05) | 0.126 |
| 7th decile | 1.05 (0.68–1.60) | 0.838 | 1.04 (0.65–1.67) | 0.874 | 1.01 (0.81–1.26) | 0.934 | 0.95 (0.75–1.21) | 0.674 |
| 8th decile | 0.82 (0.53–1.29) | 0.393 | 0.83 (0.51–1.36) | 0.458 | 0.95 (0.75–1.19) | 0.651 | 0.92 (0.72–1.17) | 0.475 |
| 9th decile | 1.01 (0.65–1.57) | 0.968 | 1.14 (0.70–1.88) | 0.592 | 1.14 (0.91–1.42) | 0.251 | 1.16 (0.91–1.47) | 0.224 |
| Highest decile | 0.74 (0.44–1.25) | 0.263 | 0.71 (0.40–1.26) | 0.242 | 1.01 (0.80–1.28) | 0.929 | 1.00 (0.78–1.29) | 0.999 |
| Per one-standard-deviation increase | 0.95 (0.85–1.07) | 0.419 | 0.94 (0.83–1.06) | 0.315 | 1.03 (0.97–1.08) | 0.382 | 1.03 (0.97–1.09) | 0.398 |
| Per 10-decile increase | 0.89 (0.62–1.28) | 0.536 | 0.81 (0.54–1.22) | 0.319 | 1.08 (0.90–1.29) | 0.415 | 1.08 (0.90–1.31) | 0.409 |
| **Genetic risk score for schizophrenia** |  |  |  |  |  |  |  |  |
| Lowest decile | 1 (ref) |  | 1 (ref) |  | 1 (ref) |  | 1 (ref) |  |
| 2nd decile | 1.00 (0.64–1.56) | 0.995 | 1.02 (0.63–1.66) | 0.927 | 0.93 (0.74–1.17) | 0.541 | 0.92 (0.72–1.17) | 0.483 |
| 3rd decile | 1.09 (0.69–1.70) | 0.722 | 1.11 (0.67–1.82) | 0.693 | 0.98 (0.78–1.24) | 0.881 | 1.03 (0.80–1.31) | 0.841 |
| 4th decile | 1.26 (0.82–1.93) | 0.296 | 1.36 (0.85–2.19) | 0.199 | 1.05 (0.83–1.32) | 0.712 | 1.08 (0.84–1.38) | 0.551 |
| 5th decile | 0.88 (0.55–1.39) | 0.576 | 0.83 (0.50–1.37) | 0.463 | 1.02 (0.81–1.27) | 0.898 | 1.01 (0.80–1.29) | 0.914 |
| 6th decile | 0.86 (0.54–1.37) | 0.519 | 0.87 (0.52–1.46) | 0.606 | 1.10 (0.87–1.38) | 0.423 | 1.14 (0.90–1.46) | 0.273 |
| 7th decile | 1.20 (0.78–1.85) | 0.412 | 1.11 (0.69–1.80) | 0.667 | 0.97 (0.78–1.22) | 0.821 | 0.96 (0.75–1.21) | 0.708 |
| 8th decile | 0.98 (0.62–1.54) | 0.933 | 0.84 (0.51–1.39) | 0.505 | 1.20 (0.96–1.50) | 0.114 | 1.21 (0.95–1.53) | 0.116 |
| 9th decile | 1.37 (0.87–2.14) | 0.173 | 1.19 (0.73–1.95) | 0.487 | 1.16 (0.93–1.44) | 0.195 | 1.16 (0.92–1.47) | 0.218 |
| Highest decile | 1.37 (0.81–2.32) | 0.247 | 1.16 (0.64–2.09) | 0.622 | 1.04 (0.79–1.37) | 0.791 | 0.99 (0.74–1.32) | 0.922 |
| Per one-standard-deviation increase | 1.08 (0.95–1.24) | 0.252 | 1.01 (0.87–1.17) | 0.939 | 1.06 (0.99–1.13) | 0.084 | 1.05 (0.98–1.13) | 0.170 |
| Per 10-decile increase | 1.25 (0.85–1.82) | 0.255 | 0.99 (0.66–1.51) | 0.980 | 1.22 (1.01–1.47) | 0.039 | 1.19 (0.97–1.45) | 0.092 |

^1^ Calendar birth year of the woman and the first 4 principal components were adjusted in the crude and adjusted odds ratio.

^2^ Further adjusted for parental psychiatric history, parental country of origin, age and age squared at the index delivery, and primiparous.

**Table S8.** Sanity check on odds ratio of ***any psychiatric disorders*** by the genetic risk scores measured continuously and in deciles with reference to the lowest decile-panel (N=479+2542+3989 vs 1840)

| **Genetic risk scores** | **Crude OR^1^** | **p-value** | **Adjusted OR^1,2^** | **p-value** |
| --- | --- | --- | --- | --- |
| **Genetic risk score for major depressive disorder** |  |  |  |  |
| Lowest decile | 1 (ref) |  | 1 (ref) |  |
| 2nd decile | 1.19 (0.94–1.50) | 0.153 | 1.16 (0.91–1.47) | 0.221 |
| 3rd decile | 1.62 (1.27–2.06) | <0.001 | 1.62 (1.27–2.07) | <0.001 |
| 4th decile | 1.41 (1.12–1.78) | 0.004 | 1.37 (1.08–1.74) | 0.009 |
| 5th decile | 1.94 (1.53–2.45) | <0.001 | 1.85 (1.45–2.35) | <0.001 |
| 6th decile | 1.62 (1.29–2.04) | <0.001 | 1.56 (1.23–1.98) | <0.001 |
| 7th decile | 1.88 (1.50–2.37) | <0.001 | 1.82 (1.44–2.30) | <0.001 |
| 8th decile | 2.16 (1.71–2.73) | <0.001 | 2.05 (1.61–2.60) | <0.001 |
| 9th decile | 2.48 (1.97–3.13) | <0.001 | 2.33 (1.84–2.95) | <0.001 |
| Highest decile | 2.66 (2.10–3.38) | <0.001 | 2.50 (1.96–3.19) | <0.001 |
| Per one-standard-deviation increase | 1.33 (1.26–1.40) | <0.001 | 1.30 (1.23–1.37) | <0.001 |
| Per 10-decile increase | 2.66 (2.21–3.19) | <0.001 | 2.48 (2.06–2.99) | <0.001 |
| **Genetic risk score for bipolar disorder** |  |  |  |  |
| Lowest decile | 1 (ref) |  | 1 (ref) |  |
| 2nd decile | 1.13 (0.91–1.42) | 0.272 | 1.14 (0.91–1.43) | 0.259 |
| 3rd decile | 1.00 (0.80–1.25) | 0.979 | 1.01 (0.80–1.26) | 0.961 |
| 4th decile | 1.13 (0.90–1.44) | 0.295 | 1.11 (0.87–1.42) | 0.384 |
| 5th decile | 1.05 (0.84–1.33) | 0.651 | 1.03 (0.82–1.30) | 0.794 |
| 6th decile | 1.22 (0.97–1.54) | 0.082 | 1.19 (0.94–1.50) | 0.146 |
| 7th decile | 1.14 (0.91–1.43) | 0.245 | 1.11 (0.89–1.40) | 0.356 |
| 8th decile | 1.02 (0.81–1.28) | 0.876 | 0.98 (0.78–1.23) | 0.841 |
| 9th decile | 1.28 (1.02–1.62) | 0.036 | 1.27 (1.00–1.61) | 0.048 |
| Highest decile | 1.35 (1.06–1.74) | 0.017 | 1.32 (1.03–1.70) | 0.031 |
| Per one-standard-deviation increase | 1.07 (1.01–1.14) | 0.016 | 1.06 (1.00–1.13) | 0.045 |
| Per 10-decile increase | 1.25 (1.04–1.50) | 0.020 | 1.20 (0.99–1.45) | 0.061 |
| **Genetic risk score for schizophrenia** |  |  |  |  |
| Lowest decile | 1 (ref) |  | 1 (ref) |  |
| 2nd decile | 1.04 (0.83–1.30) | 0.751 | 1.03 (0.82–1.29) | 0.795 |
| 3rd decile | 1.17 (0.93–1.48) | 0.171 | 1.17 (0.92–1.48) | 0.197 |
| 4th decile | 1.03 (0.82–1.29) | 0.803 | 1.05 (0.83–1.32) | 0.709 |
| 5th decile | 1.14 (0.91–1.43) | 0.255 | 1.10 (0.87–1.38) | 0.431 |
| 6th decile | 1.13 (0.90–1.42) | 0.283 | 1.15 (0.92–1.45) | 0.224 |
| 7th decile | 1.24 (0.99–1.55) | 0.061 | 1.21 (0.96–1.52) | 0.110 |
| 8th decile | 1.18 (0.94–1.48) | 0.144 | 1.13 (0.90–1.42) | 0.291 |
| 9th decile | 1.62 (1.28–2.05) | <0.001 | 1.56 (1.23–1.98) | <0.001 |
| Highest decile | 1.32 (1.00–1.75) | 0.052 | 1.17 (0.88–1.56) | 0.293 |
| Per one-standard-deviation increase | 1.16 (1.08–1.24) | <0.001 | 1.13 (1.05–1.21) | 0.001 |
| Per 10-decile increase | 1.47 (1.21–1.79) | <0.001 | 1.36 (1.12–1.66) | 0.002 |

Genetic risk scores were generated from a list of SNPs associated with major depressive disorder, bipolar disorder and schizophrenia at a p-value threshold of 0.05 or lower

^1^ Calendar birth year of the woman and the first 4 principal components were adjusted in the crude and adjusted odds ratio.

^2^ Further adjusted for parental psychiatric history, parental country of origin, age and age squared at the index delivery, and primiparous.

**Table S9.** Odds ratio of postpartum psychiatric disorders by the genetic risk scores measured continuously and in deciles with reference to the lowest decile-panel, by previous psychiatric history (with p_t_≤1.0)

|  | **Women with no psychiatric history** | | | | **Women with psychiatric history** | | | |
| --- | --- | --- | --- | --- | --- | --- | --- | --- |
| **Genetic risk scores** | Crude OR  (95% CI)^1^ | p-value | Adjusted OR (95% CI)^1,2^ | p-value | Crude OR  (95% CI)^1^ | p-value | Adjusted OR (95% CI)^1,2^ | p-value |
| **Genetic risk score for major depressive disorder** |  |  |  |  |  |  |  |  |
| Lowest decile | 1 (ref) |  | 1 (ref) |  | 1 (ref) |  | 1 (ref) |  |
| 2nd decile | 1.71 (1.01–2.90) | 0.045 | 1.32 (0.74–2.35) | 0.347 | 0.90 (0.69–1.19) | 0.481 | 0.85 (0.63–1.14) | 0.266 |
| 3rd decile | 1.38 (0.80–2.37) | 0.245 | 1.12 (0.62–2.02) | 0.710 | 1.22 (0.93–1.59) | 0.147 | 1.12 (0.84–1.48) | 0.446 |
| 4th decile | 1.59 (0.93–2.71) | 0.087 | 1.30 (0.72–2.32) | 0.384 | 1.14 (0.88–1.49) | 0.327 | 1.04 (0.79–1.38) | 0.777 |
| 5th decile | 2.12 (1.28–3.52) | 0.004 | 1.49 (0.86–2.59) | 0.153 | 1.08 (0.83–1.40) | 0.590 | 1.00 (0.76–1.33) | 0.984 |
| 6th decile | 2.59 (1.57–4.26) | <0.001 | 1.87 (1.08–3.23) | 0.025 | 1.11 (0.85–1.44) | 0.440 | 0.98 (0.74–1.30) | 0.890 |
| 7th decile | 2.36 (1.45–3.86) | 0.001 | 1.67 (0.98–2.86) | 0.060 | 1.04 (0.80–1.36) | 0.745 | 0.98 (0.74–1.29) | 0.877 |
| 8th decile | 2.10 (1.26–3.50) | 0.004 | 1.59 (0.91–2.78) | 0.102 | 1.35 (1.05–1.74) | 0.018 | 1.18 (0.91–1.54) | 0.216 |
| 9th decile | 2.55 (1.54–4.22) | <0.001 | 1.64 (0.94–2.84) | 0.082 | 1.34 (1.05–1.72) | 0.020 | 1.22 (0.94–1.58) | 0.143 |
| Highest decile | 2.47 (1.49–4.11) | <0.001 | 1.48 (0.84–2.58) | 0.173 | 1.45 (1.13–1.86) | 0.003 | 1.26 (0.97–1.63) | 0.087 |
| Per one-standard-deviation increase | 1.29 (1.16–1.43) | <0.001 | 1.14 (1.01–1.29) | 0.033 | 1.14 (1.08–1.20) | <0.001 | 1.10 (1.04–1.16) | 0.001 |
| Per 10-decile increase | 2.76 (1.56–4.88) | 0.001 | 1.58 (0.83–3.03) | 0.164 | 1.51 (1.15–1.99) | 0.003 | 1.32 (0.99–1.77) | 0.059 |
| **Genetic risk score for bipolar disorder** |  |  |  |  |  |  |  |  |
| Lowest decile | 1 (ref) |  | 1 (ref) |  | 1 (ref) |  | 1 (ref) |  |
| 2nd decile | 0.86 (0.55–1.36) | 0.530 | 0.94 (0.57–1.56) | 0.814 | 1.21 (0.96–1.52) | 0.109 | 1.19 (0.94–1.52) | 0.156 |
| 3rd decile | 1.02 (0.65–1.60) | 0.925 | 1.35 (0.82–2.21) | 0.236 | 1.17 (0.93–1.48) | 0.170 | 1.14 (0.89–1.45) | 0.293 |
| 4th decile | 1.48 (0.96–2.28) | 0.073 | 1.57 (0.98–2.53) | 0.062 | 1.09 (0.87–1.36) | 0.459 | 1.11 (0.87–1.41) | 0.396 |
| 5th decile | 0.92 (0.58–1.44) | 0.705 | 1.03 (0.62–1.70) | 0.908 | 1.20 (0.96–1.51) | 0.114 | 1.19 (0.93–1.52) | 0.161 |
| 6th decile | 0.88 (0.55–1.39) | 0.580 | 1.03 (0.62–1.70) | 0.923 | 1.19 (0.95–1.49) | 0.135 | 1.16 (0.91–1.48) | 0.217 |
| 7th decile | 1.00 (0.64–1.57) | 0.999 | 1.10 (0.67–1.81) | 0.707 | 1.06 (0.84–1.33) | 0.632 | 1.03 (0.81–1.31) | 0.829 |
| 8th decile | 1.04 (0.67–1.63) | 0.848 | 1.01 (0.62–1.66) | 0.959 | 0.98 (0.78–1.23) | 0.857 | 0.97 (0.76–1.24) | 0.825 |
| 9th decile | 1.15 (0.74–1.78) | 0.539 | 1.25 (0.77–2.04) | 0.362 | 1.07 (0.85–1.34) | 0.559 | 1.08 (0.85–1.37) | 0.542 |
| Highest decile | 1.30 (0.79–2.16) | 0.305 | 1.38 (0.79–2.43) | 0.257 | 1.21 (0.94–1.54) | 0.139 | 1.20 (0.92–1.56) | 0.185 |
| Per one-standard-deviation increase | 1.04 (0.92–1.17) | 0.573 | 1.02 (0.89–1.17) | 0.806 | 1.02 (0.96–1.08) | 0.617 | 1.02 (0.95–1.08) | 0.625 |
| Per 10-decile increase | 1.18 (0.81–1.70) | 0.390 | 1.12 (0.74–1.69) | 0.586 | 0.97 (0.81–1.17) | 0.757 | 0.97 (0.80–1.18) | 0.774 |
| **Genetic risk score for schizophrenia** |  |  |  |  |  |  |  |  |
| Lowest decile | 1 (ref) |  | 1 (ref) |  | 1 (ref) |  | 1 (ref) |  |
| 2nd decile | 1.26 (0.81–1.96) | 0.306 | 1.26 (0.77–2.04) | 0.354 | 1.06 (0.84–1.33) | 0.625 | 1.07 (0.84–1.37) | 0.566 |
| 3rd decile | 1.23 (0.79–1.91) | 0.353 | 1.22 (0.75–1.99) | 0.418 | 0.97 (0.77–1.22) | 0.800 | 1.05 (0.82–1.34) | 0.690 |
| 4th decile | 1.04 (0.66–1.63) | 0.857 | 1.00 (0.61–1.65) | 0.988 | 1.04 (0.83–1.31) | 0.705 | 1.07 (0.84–1.36) | 0.580 |
| 5th decile | 1.08 (0.69–1.68) | 0.749 | 1.01 (0.62–1.65) | 0.978 | 1.07 (0.85–1.33) | 0.575 | 1.10 (0.87–1.40) | 0.418 |
| 6th decile | 1.28 (0.62–1.55) | 0.272 | 1.19 (0.73–1.94) | 0.477 | 1.12 (0.90–1.40) | 0.297 | 1.11 (0.88–1.39) | 0.398 |
| 7th decile | 0.98 (0.62–1.55) | 0.937 | 0.81 (0.48–1.34) | 0.407 | 1.07 (0.85–1.34) | 0.556 | 1.12 (0.89–1.43) | 0.333 |
| 8th decile | 1.10 (0.71–1.71) | 0.666 | 0.90 (0.55–1.45) | 0.662 | 1.10 (0.88–1.37) | 0.391 | 1.16 (0.92–1.46) | 0.214 |
| 9th decile | 1.62 (1.04–2.50) | 0.032 | 1.31 (0.81–2.12) | 0.276 | 1.05 (0.84–1.30) | 0.682 | 1.06 (0.84–1.34) | 0.604 |
| Highest decile | 0.99 (0.54–1.81) | 0.980 | 0.81 (0.42–1.59) | 0.549 | 1.33 (1.00–1.76) | 0.049 | 1.27 (0.94–1.71) | 0.118 |
| Per one-standard-deviation increase | 1.06 (0.92–1.22) | 0.438 | 0.95 (0.81–1.12) | 0.563 | 1.05 (0.98–1.13) | 0.189 | 1.04 (0.97–1.13) | 0.267 |
| Per 10-decile increase | 1.15 (0.79–1.68) | 0.475 | 0.86 (0.57–1.32) | 0.495 | 1.15 (0.95–1.39) | 0.140 | 1.14 (0.94–1.39) | 0.191 |

Genetic risk scores were generated from a list of SNPs associated with major depressive disorder, bipolar disorder and schizophrenia at a p-value threshold of 1 or lower

^1^ Calendar birth year of the woman and the first 4 principal components were adjusted in the crude and adjusted odds ratio.

^2^ Further adjusted for parental psychiatric history, parental country of origin, age and age squared at the index delivery, and parity.

**Table S10** The odds ratio of postpartum psychiatric disorders by the genetic risk scores and previous psychiatric history among women born to Danish parents (n=8 026)

| **Genetic risk scores** | **Women with no psychiatric history** | | | | **Women with psychiatric history** | | | |
| --- | --- | --- | --- | --- | --- | --- | --- | --- |
|  | **Crude OR**  **(95% CI)^1^** | **p-value** | **Adjusted OR (95% CI)^1,2^** | **p-value** | **Crude OR**  **(95% CI)^1^** | **p-value** | **Adjusted OR (95% CI)^1,2^** | **p-value** |
| **Genetic risk score for major depression** |  |  |  |  |  |  |  |  |
| Per one-standard-deviation increase | 1.31 (1.17–1.46) | <0.001 | 1.17 (1.04–1.32) | 0.011 | 1.13 (1.07–1.19) | <0.001 | 1.08 (1.02–1.15) | 0.007 |
| Per 10-decile increase | 2.52 (1.73–3.68) | <0.001 | 1.83 (1.20–2.78) | 0.005 | 1.56 (1.29–1.88) | <0.001 | 1.39 (1.15–1.70) | 0.001 |
| **Genetic risk score for bipolar disorder** |  |  |  |  |  |  |  |  |
| Per one-standard-deviation increase | 0.92 (0.81–1.04) | 0.168 | 0.91 (0.80–1.04) | 0.170 | 1.02 (0.96–1.08) | 0.498 | 1.02 (0.96–1.08) | 0.570 |
| Per 10-decile increase | 0.80 (0.55–1.17) | 0.255 | 0.74 (0.49–1.14) | 0.172 | 1.07 (0.89–1.29) | 0.471 | 1.07 (0.88–1.31) | 0.494 |
| **Genetic risk score for schizophrenia** |  |  |  |  |  |  |  |  |
| Per one-standard-deviation increase | 1.04 (0.90–1.19) | 0.613 | 0.98 (0.84–1.14) | 0.778 | 1.06 (0.99–1.14) | 0.099 | 1.05 (0.98–1.13) | 0.192 |
| Per 10-decile increase | 1.09 (0.74–1.62) | 0.657 | 0.91 (0.59–1.40) | 0.676 | 1.19 (0.98–1.45) | 0.076 | 1.16 (0.95–1.42) | 0.156 |

^1^ Calendar birth year of the woman and the first 4 principal components were adjusted in the crude and adjusted odds ratio.

^2^ Further adjusted for parental psychiatric history, age and age squared at the index delivery, and primiparous.

**Table S11a** Odds ratio of postpartum psychiatric disorders ***defined by hospital contact*** by the genetic risk scores and previous psychiatric history (N=7117)

| **Genetic risk scores** | **Women with no psychiatric history** | | | | **Women with psychiatric history** | | | |
| --- | --- | --- | --- | --- | --- | --- | --- | --- |
|  | **Crude OR**  **(95% CI)** | **p-value** | **Adjusted OR (95% CI) *** | **p-value** | **Crude OR**  **(95% CI)** | **p-value** | **Adjusted OR (95% CI) *** | **p-value** |
| **Genetic risk score for major depression** |  |  |  |  |  |  |  |  |
| Per one-standard-deviation increase | 1.26 (1.10–1.43) | 0.001 | 1.14 (0.99–1.31) | 0.061 | 1.16 (1.08–1.25) | <0.001 | 1.10 (1.02–1.19) | 0.019 |
| Per 10-decile increase | 2.19 (1.41–3.41) | <0.001 | 1.72 (1.07–2.78) | 0.026 | 1.67 (1.30–2.16) | <0.001 | 1.39 (1.06–1.83) | <0.001 |
| **Genetic risk score for bipolar disorder** |  |  |  |  |  |  |  |  |
| Per one-standard-deviation increase | 1.02 (0.89–1.17) | 0.798 | 1.00 (0.86–1.16) | 0.995 | 1.04 (0.96–1.12) | 0.302 | 1.04 (0.96–1.13) | 0.367 |
| Per 10-decile increase | 1.10 (0.71–1.73) | 0.665 | 1.01 (0.62–1.63) | 0.981 | 1.14 (0.89–1.47) | 0.289 | 1.14 (0.87–1.48) | 0.350 |
| **Genetic risk score for schizophrenia** |  |  |  |  |  |  |  |  |
| Per one-standard-deviation increase | 1.10 (0.93–1.29) | 0.274 | 1.01 (0.85–1.21) | 0.869 | 1.06 (0.97–1.17) | 0.213 | 1.05 (0.95–1.16) | 0.322 |
| Per 10-decile increase | 1.30 (0.81–2.06) | 0.275 | 1.03 (0.63–1.70) | 0.895 | 1.22 (0.94–1.59) | 0.127 | 1.20 (0.91–1.58) | 0.201 |

^1^ Calendar birth year of the woman and the first 4 principal components were adjusted in the crude and adjusted odds ratio.

^2^ Further adjusted for parental psychiatric history, age and age squared at the index delivery, and primiparous.

Pseudo-R^2^=0.29% for genetic risk score of major depression, 0.00% for bipolar disorder and 0.00% for schizophrenia among women with no previous psychiatric history. Pseudo-R^2^=0.11% for major depression, 0.02% for bipolar disorder and 0.03% for schizophrenia among women with previous psychiatric history.

**Table S11b** Odds ratio of postpartum psychiatric disorders ***defined by psychotropic prescription only*** by the genetic risk scores and previous psychiatric history (N=7565)

| **Genetic risk scores** | **Women with no psychiatric history** | | | | **Women with psychiatric history** | | | |
| --- | --- | --- | --- | --- | --- | --- | --- | --- |
|  | **Crude OR**  **(95% CI)** | **p-value** | **Adjusted OR (95% CI) *** | **p-value** | **Crude OR**  **(95% CI)** | **p-value** | **Adjusted OR (95% CI) *** | **p-value** |
| **Genetic risk score for major depression** |  |  |  |  |  |  |  |  |
| Per one-standard-deviation increase | 1.39 (1.18–1.62) | <0.001 | 1.22 (1.03–1.44) | 0.022 | 1.12 (1.05–1.19) | <0.001 | 1.09 (1.02–1.16) | 0.010 |
| Per 10-decile increase | 3.12 (1.81–5.37) | <0.001 | 2.13 (1.19–3.82) | 0.011 | 1.52 (1.23–1.88) | <0.001 | 1.42 (1.14–1.77) | 0.002 |
| **Genetic risk score for bipolar disorder** |  |  |  |  |  |  |  |  |
| Per one-standard-deviation increase | 0.86 (0.72–1.02) | 0.085 | 0.86 (0.72–1.03) | 0.107 | 1.02 (0.95–1.09) | 0.636 | 1.01 (0.95–1.09) | 0.682 |
| Per 10-decile increase | 0.64 (0.37–1.10) | 0.107 | 0.62 (0.34–1.11) | 0.109 | 1.04 (0.85–1.29) | 0.687 | 1.04 (0.84–1.30) | 0.705 |
| **Genetic risk score for schizophrenia** |  |  |  |  |  |  |  |  |
| Per one-standard-deviation increase | 1.06 (0.86–1.30) | 0.582 | 0.99 (0.80–1.23) | 0.952 | 1.06 (0.98–1.15) | 0.161 | 1.04 (0.96–1.13) | 0.309 |
| Per 10-decile increase | 1.19 (0.67–2.09) | 0.558 | 0.97 (0.53–1.77) | 0.923 | 1.21 (0.97–1.51) | 0.092 | 1.17 (0.93–1.47) | 0.189 |

^1^ Calendar birth year of the woman and the first 4 principal components were adjusted in the crude and adjusted odds ratio.

^2^ Further adjusted for parental psychiatric history, age and age squared at the index delivery, and primiparous.

Pseudo-R^2^=0.43% for genetic risk score of major depression, 0.21% for bipolar disorder and 0.00% for schizophrenia among women with no previous psychiatric history. Pseudo-R^2^=0.14% for major depression, 0.00% for bipolar disorder and 0.02% for schizophrenia among women with previous psychiatric history.

**Figure S1.** Covariate-adjusted receiver operating characteristic curves for major depression genetic risk score in distinguishing between postpartum psychiatric disorder cases and controls, by personal psychiatric history


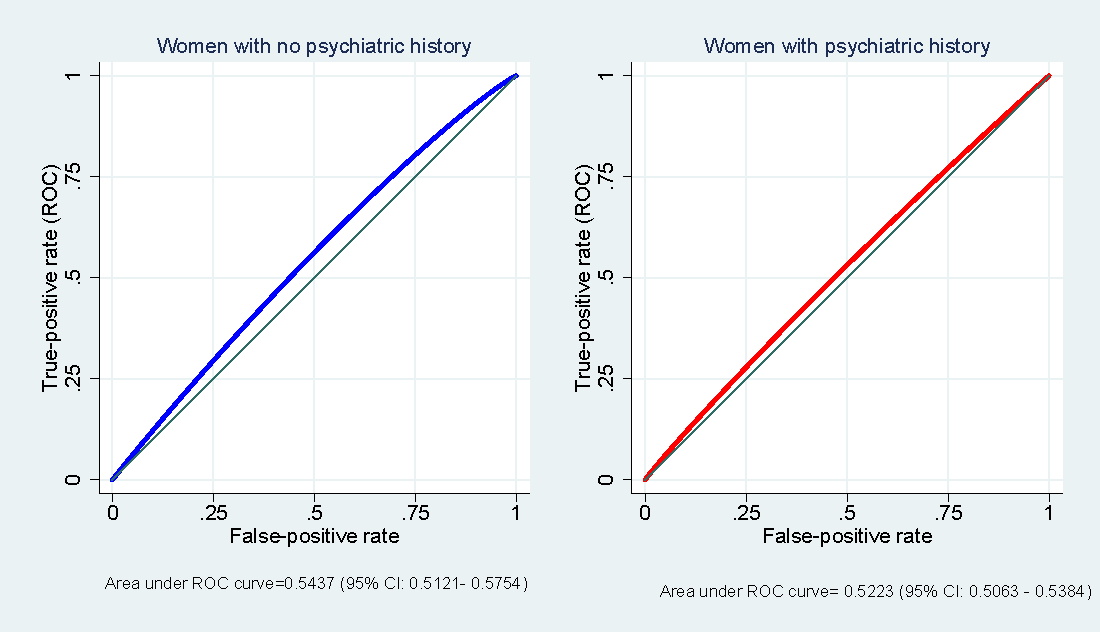


Adjusted for calendar birth year of the woman, the first 4 principal components, parental psychiatric history, parental country of origin, age and age squared at the index delivery, and primiparous. The 95% confidence interval of area under the ROC curve was calculated using bootstrap.

**Figure S2.** Covariate-adjusted receiver operating characteristic curves for bipolar disorder genetic risk score in distinguishing between postpartum psychiatric disorder cases and controls, by personal psychiatric history


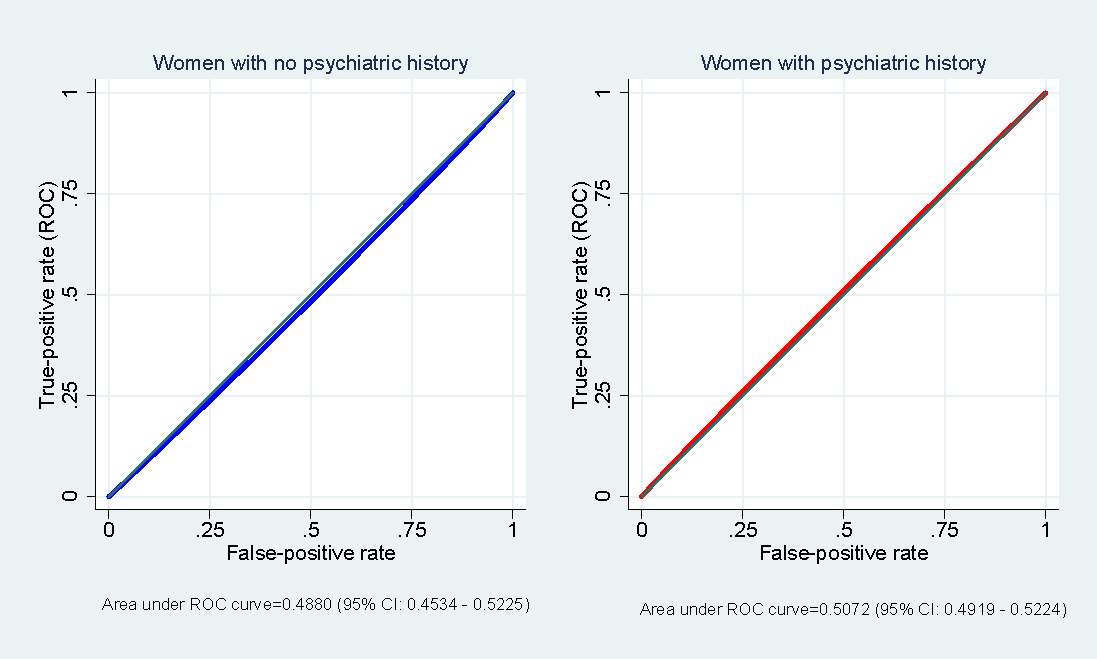


Adjusted for calendar birth year of the woman, the first 4 principal components, parental psychiatric history, parental country of origin, age and age squared at the index delivery, and primiparous. The 95% confidence interval of area under the ROC curve was calculated using bootstrap.

**Figure S3.** Covariate-adjusted receiver operating characteristic curves for schizophrenia genetic risk score in distinguishing between postpartum psychiatric disorder cases and controls, by personal psychiatric history


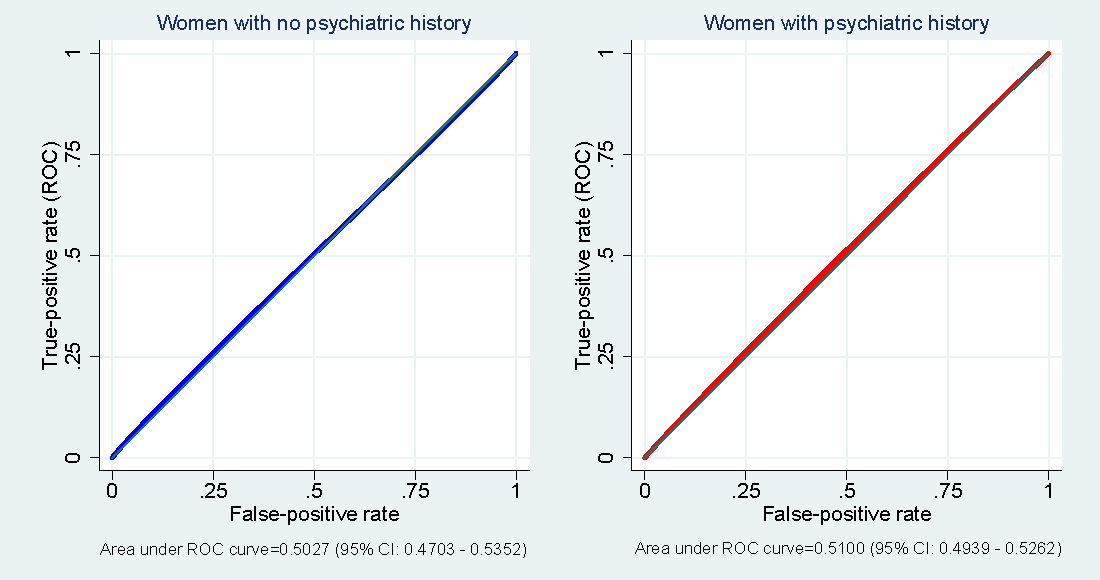


Adjusted for calendar birth year of the woman, the first 4 principal components, parental psychiatric history, parental country of origin, age and age squared at the index delivery, and primiparous. The 95% confidence interval of area under the ROC curve was calculated using bootstrap.
